# Platelet-specific SLFN14 deletion causes macrothrombocytopenia and platelet dysfunction through dysregulated megakaryocyte and platelet gene expression

**DOI:** 10.1172/JCI189100

**Published:** 2025-08-12

**Authors:** Rachel J. Stapley, Xenia Sawkulycz, Gabriel H.M. Araujo, Maximilian Englert, Lourdes Garcia-Quintanilla, Sophie R.M. Smith, Amna Ahmed, Elizabeth J. Haining, Nayandeep Kaur, Andrea Bacon, Andrey V. Pisarev, Natalie S. Poulter, Dean Kavanagh, Steven G. Thomas, Samantha J. Montague, Julie Rayes, Zoltan Nagy, Neil V. Morgan

**Affiliations:** 1 Department of Cardiovascular Sciences, School of Medical Sciences, College of Medicine and Health, University of Birmingham, Edgbaston, United Kingdom.; 2 Institute of Experimental Biomedicine, University Hospital Würzburg, Würzburg, Germany.; 3 MRC Centre for Immune Regulation, Transgenics Facility, College of Medical and Dental Sciences, University of Birmingham, Edgbaston, United Kingdom.; 4 Department of Biochemistry and Molecular Pharmacology, NYU Grossman School of Medicine, New York, New York, USA.

**Keywords:** Genetics, Hematology, Mouse models, Platelets, Thrombin

## Abstract

Schlafen 14–related (SLFN14-related) thrombocytopenia is a rare bleeding disorder caused by *SLFN14* mutations altering hemostasis in patients with platelet dysfunction. SLFN proteins are highly conserved in mammals where SLFN14 is specifically expressed in megakaryocyte (MK) and erythroblast lineages. The role of *SLFN14* in megakaryopoiesis and platelet function has not been elucidated. Therefore, we generated a murine model with a platelet- and MK-specific *SLFN14* deletion using platelet factor 4 (PF4) Cre-mediated deletion of exons 2 and 3 in *Slfn14* (*Slfn14 PF4-Cre*) to decipher the molecular mechanisms driving the bleeding phenotype. *Slfn14 PF4-Cre^+^* platelets displayed reduced platelet signaling to thrombin, reduced thrombin formation, increased bleeding tendency, and delayed thrombus formation as assessed by intravital imaging. Moreover, fewer in situ bone marrow MKs were present compared with controls. RNA-Seq and Gene Ontology analysis of MKs and platelets from *Slfn14 PF4-Cre* homozygous mice revealed altered pathways of ubiquitination, adenosine triphosphate activity, and cytoskeleton and molecular function. In summary, we investigated how SLFN14 deletion in MKs and platelets leads to platelet dysfunction and alters their transcriptome, explaining the platelet dysfunction and bleeding in humans and mice with *SLFN14* mutations.

## Abstract 2

Mutations in SLFN14 cause bleeding, low platelets and dysfunction in humans. We made a new mouse model to explain the human condition and the bleeding.

## Graphical abstract

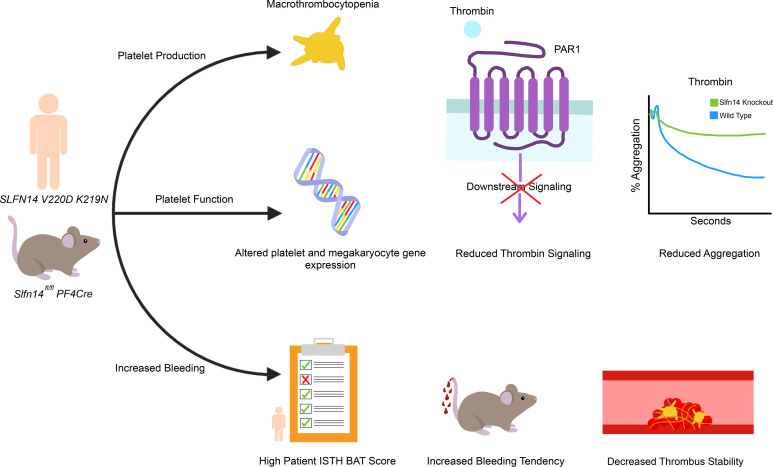

## Introduction

The Schlafen (*SLFN*) family of genes and proteins have recently been described as species-specific regulators in lineage commitment involving erythrocytes and platelets (1). SLFN14 acts as an endoribonuclease involved in the cleavage of RNA, which regulates translation (2). We previously reported 3 unrelated families with missense mutations in *SLFN14* and an associated inherited bleeding disorder through the UK Genotyping and Phenotyping of Platelets (GAPP) study (3, 4). Initial functional investigation of the patients with *SLFN14* mutations (V220D, K218E, and K219N) revealed mild macrothrombocytopenia; platelet function defects in response to adenosine diphosphate (ADP), collagen, and protease-activated receptor 1 (PAR1) peptide; and decreased adenosine triphosphate (ATP) secretion (3). Subsequent patient cohort sequencing studies have identified additional patients with mutations in *SLFN14* (5–8). Further in vitro investigation in rabbit reticulocytes and HEK293T cells revealed that SLFN14 colocalizes with ribosomes and cleaves RNA (preferentially rRNA) and ribosome-associated mRNA, resulting in endoribonucleolytically mediated degradation of RNA (2, 9). A subsequent study linked *SLFN14* pathogenic variants to dysregulated mTORC1 signaling (10). Collectively, these findings suggest that SLFN14 is critical in the proliferation of cells and transcription of mediators of signaling in hematopoiesis.

In our previous study, we published results from a global CRISPR-mediated knockin *Slfn14* mouse model (K208N), the mouse homolog of the patient K219N mutation (1). The data showed that homozygous *Slfn14* K208N resulted in a largely erythrocyte phenotype with microcytic erythrocytosis, with homozygous embryonic lethality beyond day 16.5 in utero. Heterozygous mice presented with microcytic erythrocytosis, severe anemia, splenomegaly, and abnormal thrombus formation in vivo (1). Unlike the human *SLFN14* K219N mutation, mice did not present with a major bleeding or platelet defect (1). This suggested that SLFN14, as an endoribonuclease, plays a critical role in hematopoiesis and may function to cleave RNAs during hematopoietic stem cell fates (1).

This function at the global level may explain the involvement of SLFN14 in a species-specific manner with mutations causing platelet and erythrocyte functional differences in humans and mice, respectively. However, the mechanistic role of SLFN14 in platelet production and function remains to be explored. To address this, we generated a conditional KO mouse model using the well-established Cre/*loxP* selective deletion system with high efficiency in the megakaryocyte (MK) lineage under expression of the platelet factor 4 (PF4) promoter (11). Using this approach, we aimed to uncover the mechanism of SLFN14 within megakaryopoiesis and provide specific insights into the platelet production and functional defects observed in patients. Furthermore, we performed RNA-Seq in MKs and platelets to identify differentially expressed genes (DEGs) and pathways regulated by SLFN14 critical to the production and functionality of MKs and platelets.

## Results

### Patients with SLFN14 mutations have reduced platelet signaling response to major platelet agonists.

**Figure 1:**
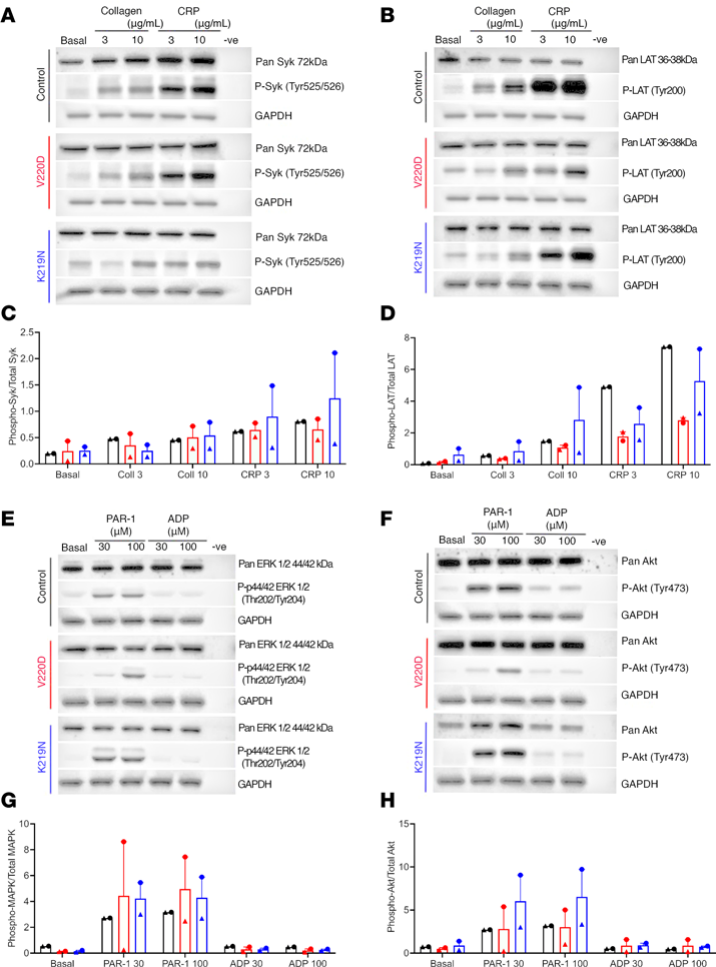
Patient-derived *SLFN14* mutant platelets show reduced signaling in response to major platelet agonists assessed by Western blotting. Major platelet agonists were used to activate platelets and assess downstream signaling of platelet receptors GPVI, PAR1, P2Y_1_, and P2Y_12_. (**A**) Platelets stimulated with low (3 μg/mL) and high doses (10 μg/mL) of collagen and CRP were used to assess P-Syk Tyr525/526. V220D and K219N patients showed reduced levels of phosphorylation compared with healthy control. (**B**) Platelets stimulated with low and high doses of collagen and CRP were used to assess P-LAT Tyr200. V220D and K219N patients showed reduced phosphorylation with increasing dose of agonist against control. (**E**) Downstream signaling of PAR1, P2Y_1_, and P2Y_12_ receptors assessed by P-ERK1/2 Tyr202/204 (MAPK) after stimulation with low and high doses of PAR1 peptide (30–100 μM) and ADP (30–100 μM). SLFN14 mutants showed reduced phosphorylation of ERK1/2 compared with control. (**F**) Reduced phosphorylation of P-AKT Tyr473 was observed in both patients with *SLFN14* mutations. (**C**, **D**, **G**, and **H**) Quantification of Western blots normalized to GAPDH housekeeping gene. The ratio of pan/phosphorylation was measured and plotted. Biological replicates shown for healthy controls and patient samples; *n* = 2. Please note that the GAPDH images for control in **A** and **B**, V220D in **A** and **B**, and control in **E** and **F** are the same across the panels as they are derived from the same samples.

We previously showed that patients with *SLFN14* mutations had reduced platelet aggregation and granule secretion. Reduced platelet activation was to classical platelet agonists including ADP, collagen-related peptide (CRP), and PAR1 peptide (3), suggesting a reduction in GPCR and tyrosine kinase signaling. Here, we investigated activation of downstream platelet signaling in several patients from the original families with *SLFN14* mutations V220D and K219N (Figure 1, A–H) (3). Patient and healthy control platelets were activated with collagen, CRP, PAR1 peptide, and ADP at various doses. After CRP stimulation with both high and low doses, we observed reduced linker of activated T cell (LAT) phosphorylation in both *SLFN14* patients (V220D and K219N) compared with heathy controls, suggesting reduced immunoreceptor tyrosine-based activation motif (ITAM) signaling (Figure 1, B and D). Further, when stimulated with PAR1 peptide (30 μM), we observed lower AKT and ERK1/2 phosphorylation in the V220D mutant patients (Figure 1, E–H).

### PF4-Cre–mediated deletion of exons 2 and 3 in Slfn14 results in mild macrothrombocytopenia in mice.

**Figure 2:**
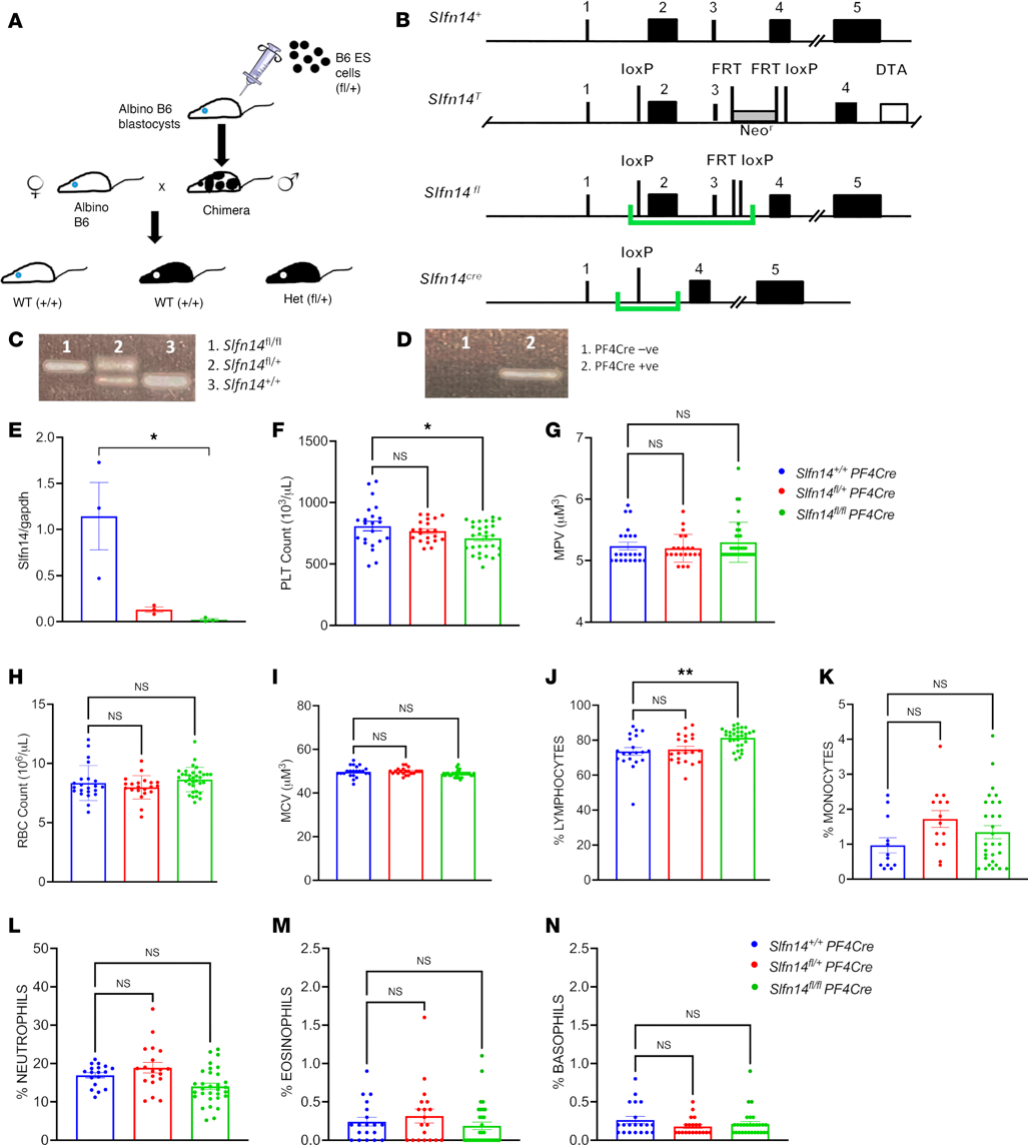
*Slfn14 PF4-Cre* mice generated using a Cre/*LoxP* conditional KO system have mild macrothrombocytopenia. (**A**) Clones (P1B8 and P1F1) were generated containing the *Slfn14* transgene. Germline testing and transmission were performed to identify different coat colors of the embryonic stem (ES) cells and the host blastocysts. (**B**) Targeting strategy used to generate the SLFN14-floxed mouse model. Green brackets highlight regions to be deleted upon Cre expression by *loxP* sites. *Slfn14*^cre^ shows *Slfn14* gene structure upon conditional KO of exons 2 and 3 after PF4-Cre recombinase activity. FRT, flippase recognition target; DTA, diphtheria toxin subunit A. (**C**) Detection of *Slfn14^fl^* alleles by PCR and agarose gel electrophoresis. Genotypes 1–3 are *Slfn14^fl/fl^*, *Slfn14^fl/+^*, and *Slfn14^+/+^*, respectively. (**D**) Detection of PF4-Cre expression by PCR and agarose gel electrophoresis. Genotypes 1 and 2 are negative and positive for PF4-Cre, respectively. (**E**) Verification of SLFN14-KO in platelets shown by lack of SLFN14 expression in *Slfn14^fl/fl^* PF4-Cre mice and reduced expression in *Slfn14^fl/+^* PF4-Cre mice using quantitative RT-PCR. Data are mean ± SEM; 3 mice per genotype; *n* = 3. *P < 0.05. (**F**) *Slfn14^fl/fl^* PF4-Cre mice show significantly reduced platelet count (*P* = 0.0012). (**G**) *Slfn14^fl/fl^* PF4-Cre mice have a tendency to have enlarged platelets (increased MPV) compared with controls but did not reach significance. Data are mean ± SEM; *n* = 21–30 mice per genotype. Significance was assessed by 1-way ANOVA with multiple comparisons. (**H**) RBC count. (**I**) Mean corpuscular volume (MCV). (**J**) *Slfn14^fl/fl^* PF4-Cre mice show significant increase in percent lymphocytes. (**K**) Percent monocytes, (**L**) percent neutrophils, (**M**) percent eosinophils, and (**N**) percent basophils in mice were consistent across all genotypes. Data are presented as mean ± SEM; 1-way ANOVA with correction for multiple comparisons. *n* = 11–30 mice per genotype.

We generated a conditional KO mouse model to evaluate the mechanistic role of *Slfn14* in platelet function (Figure 2). *LoxP* sites flanking exons 2 and 3 of *Slfn14* were inserted to create a *Slfn14^fl^* allele. Positive expression of the PF4-Cre recombinase (11) resulted in selective deletion of exons 2 and 3 in *Slfn14*, specifically in MKs and platelets (Figure 2, A and B). Heterozygote breeding of *Slfn14^fl/+^* and *Slfn14^fl/+^* PF4-Cre pairs resulted in the expected genotypes, which was confirmed by PCR (Figure 2, C and D). A χ^2^ analysis showed that heterozygote breeding produced offspring in ratios according to Mendelian inheritance and did not show preferential segregation of 1 allele over another (Supplemental Figure 1). Quantitative real-time PCR (RT-PCR) revealed complete loss of *Slfn14* expression in *Slfn14^fl/fl^* PF4-Cre (homozygous) mice and approximately 50% expression in *Slfn14^fl/+^* PF4-Cre (heterozygous) mice (Figure 2E). Exon 1 in *SLFN14* is a noncoding exon; therefore, the absence of a start codon (due to deletion of exons 2 and 3) results in complete loss of SLFN14 expression in MKs and platelets. Whole-blood counting revealed that *Slfn14^fl/fl^* PF4-Cre mice present with mild thrombocytopenia (Figure 2F). While *Slfn14^fl/+^* PF4-Cre mice also show this pattern, platelet counts were not significantly different to controls, suggesting that only complete loss of *Slfn14* impacts platelet production in hematopoiesis. Mean platelet volume (MPV) measured in whole blood was not significant, though it trended toward a higher value in *Slfn14^fl/fl^* PF4-Cre mice (Figure 2G), as assessed via flow cytometry and electron microscopy analysis below. All other hematological parameters, including blood cell counts, were unchanged, aside from increased percent lymphocytes, which might be explained by PF4-Cre–driven recombination in these cells (Figure 2, H–N) (12).

### Slfn14 PF4-Cre mice have normal levels of major platelet glycoprotein receptors and increased size and granule content.

**Figure 3:**
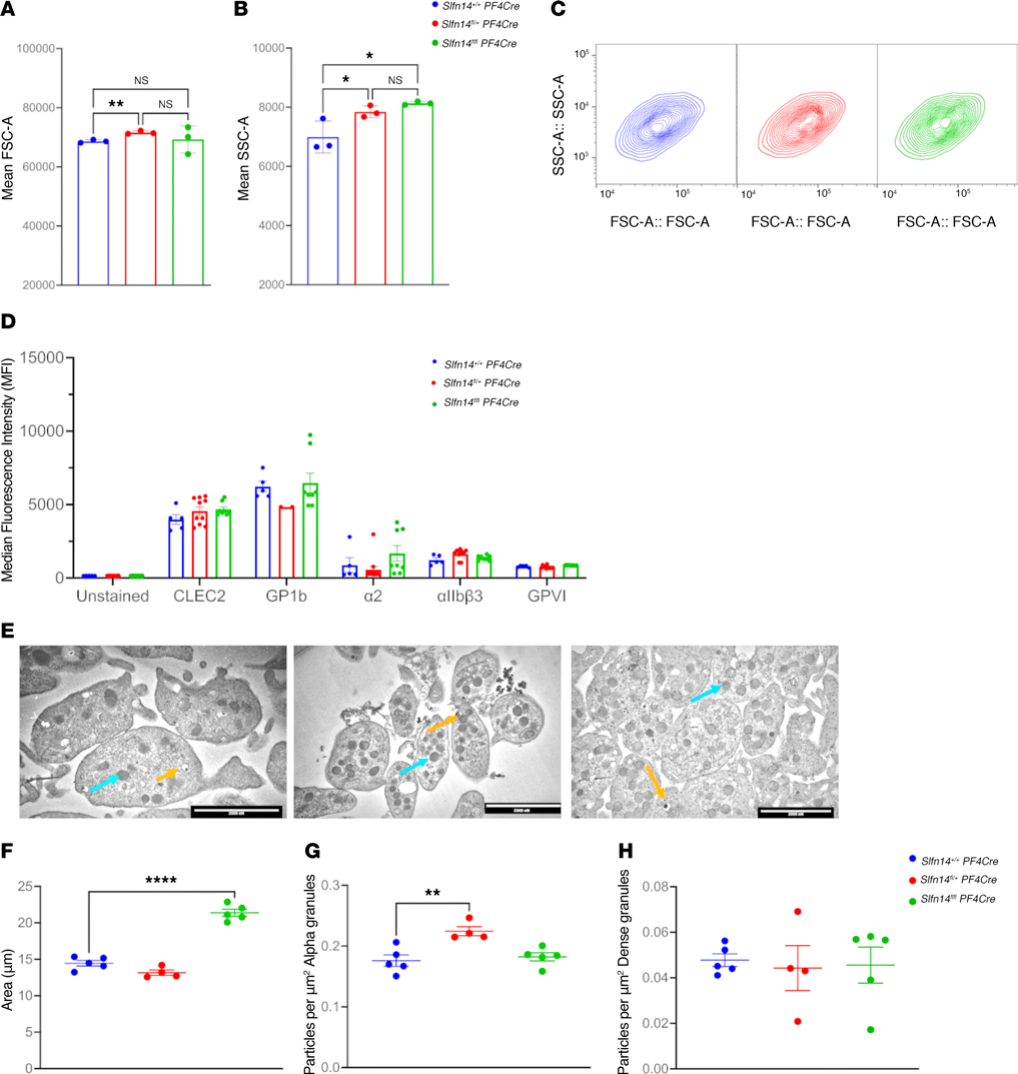
In vitro assessment of platelet size and glycoprotein levels and TEM of platelets in *Slfn14* PF4-Cre mice. (**A**–**C**) *Slfn14* PF4-Cre mice show macrothrombocytopenia. Flow cytometry confirms increased platelet size and granularity (FSC-A and SSC-A) in *Slfn14^fl/+^* PF4-Cre and *Slfn14^fl/fl^* PF4-Cre. Data are mean ± SEM; 2-way ANOVA with correction for multiple comparisons; *P < 0.05, **P < 0.01. (**D**) Resting surface glycoprotein expression levels measured by flow cytometry. GPIbα^+^ platelets were costained using indicated surface markers in a whole-blood flow cytometry assay. Data presented are MFI ± SEM from *n* = 5–11 mice per genotype; significance assessed using 2-way ANOVA with correction for multiple comparisons. (**E**) Representative TEM images of each genotype of *Slfn14* PF4-Cre platelets. Scale bars: 2,000 nm. (**F**–**H**) Quantification of area (μm^2^), α granule (blue arrows in **E**), and dense (δ) granule (orange arrows in **E**) content in *Slfn14* PF4-Cre platelets using TEM.

Using flow cytometry to estimate platelet size by forward scatter (FSC) showed slightly increased values in *Slfn14^fl/+^* PF4-Cre mice (Figure 3, A and C). In addition, platelet side scatter (SSC) revealed an increase in both *Slfn14^fl/+^* PF4-Cre and *Slfn14^fl/fl^* PF4-Cre compared with control mice (Figure 3B).In vitro assessment of surface expression of platelet glycoprotein receptors by flow cytometry revealed no significant differences between *Slfn14^fl/fl^* PF4-Cre mice and controls (Figure 3D). Resting platelet ultrastructure was assessed, and granule numbers were quantified using transmission electron microscopy (TEM) (Figure 3E). Dense (δ) granule numbers were not significantly increased in the *SLFN14^fl/fl^* PF4-Cre mice nor the *Slfn14^fl/+^* PF4-Cre mice when compared with the control, after correcting for the platelet area. However, α granules showed a significant difference in the *Slfn14^fl/+^* PF4-Cre mice compared with the control, but a similar increase was not observed for the *Slfn14^fl/fl^* PF4-Cre mice (Figure 3, F and H). However, platelet area was significantly increased in *Slfn14^fl/fl^* PF4-Cre (Figure 3G). Overall, flow cytometry and TEM analysis showed that the *Slfn14^fl/fl^* PF4-Cre and *Slfn14^fl/+^* PF4-Cre mice had markedly increased platelet size and granule content compared with control mice (Figure 3, F and G). *Slfn14* PF4-Cre platelets demonstrated no significant difference in spreading on collagen and fibrinogen in comparison with control platelets (Supplemental Figure 3).

### Slfn14^fl/fl^ PF4-Cre mice show reduced platelet aggregation and secretion in response to thrombin-associated agonists.

**Figure 4:**
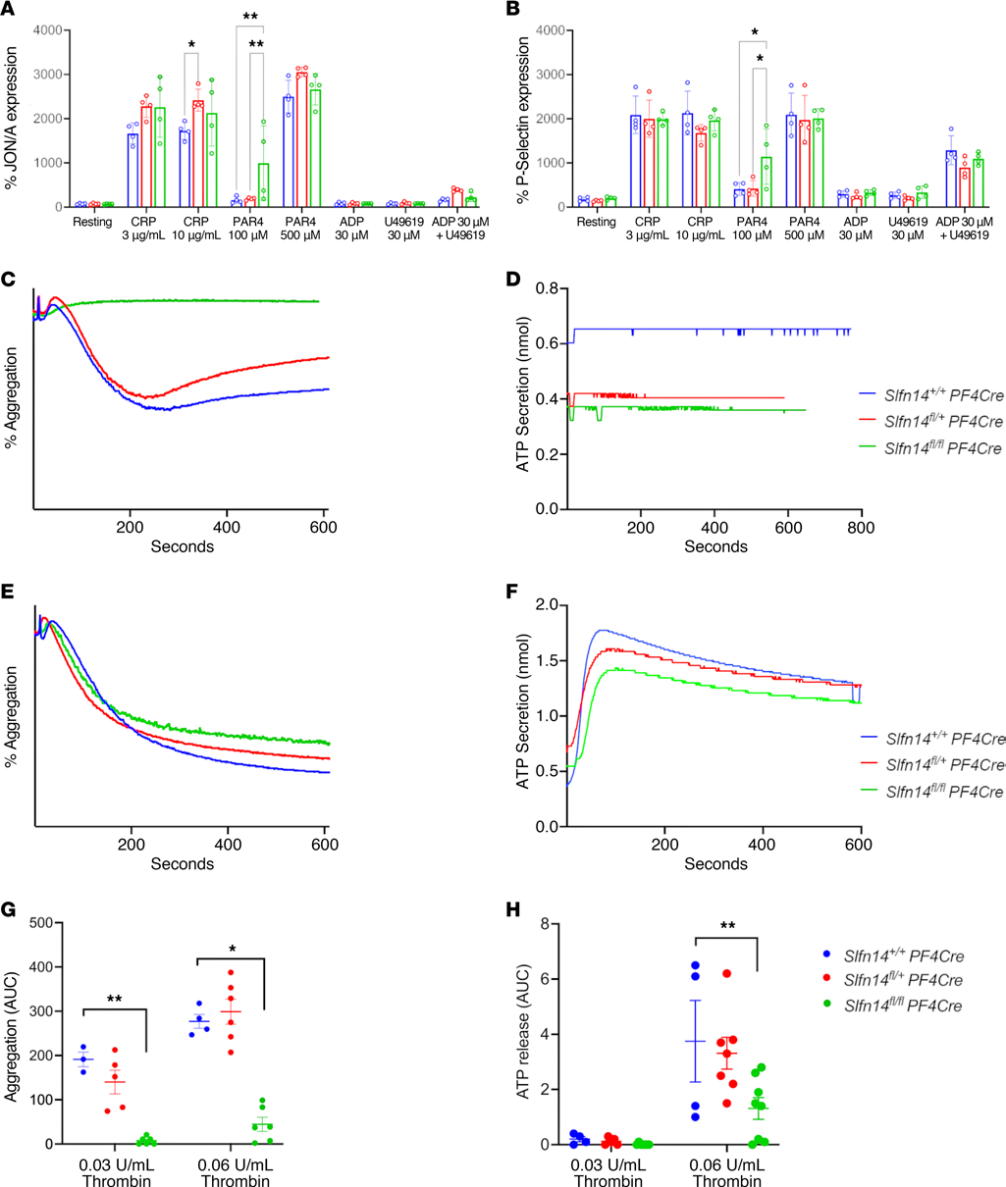
*Slfn14* PF4-Cre platelets present with reduced aggregation and secretion in response to thrombin. (**A**) Activated integrin αIIbβ3 (JON/A) and (**B**) P-selectin expression in *Slfn14* PF4-Cre mouse platelets in response to indicated agonist stimulation. *Slfn14^fl/+^* PF4-Cre and *Slfn14^fl/fl^* PF4-Cre show normal expression in response to all agonists tested. Data presented are percentage of JON/A^+^ or P-selectin^+^ expression; mean ± SEM from *n* = 5–11 mice per genotype. Significance assessed by 2-way ANOVA with correction for multiple comparisons. (**C** and **E**) Representative traces of *Slfn14* PF4-Cre aggregation in response to (**C**) 0.03 U/mL thrombin and (**E**) 0.06 U/mL thrombin. Quantified AUC for washed platelet aggregation in response to thrombin. (**D** and **F**) Representative traces of *Slfn14* PF4-Cre ATP secretion in nanomole measured by Chrono-Lume luciferin-luciferase reagent for (**D**) 0.03 U/mL thrombin and (**F**) 0.06 U/mL thrombin. (**G**) Aggregation data presented are mean AUC ± SEM, representative traces of *n* = 4–10 mice per genotype; significance was assessed by 2-way ANOVA with correction for multiple comparisons. (**H**) ATP secretion data quantification and measurement by AUC in response to thrombin. Representative traces of *n* = 4–10 mice per genotype; significance was assessed by 2-way ANOVA with correction for multiple comparisons. *P < 0.05 and **P < 0.01.

*Slfn14^fl/+^* PF4-Cre and *Slfn14^fl/fl^* PF4-Cre platelets showed normal expression levels to JON/A and P-selectin activation in response to all platelet agonists tested (Figure 4, A and B) (13). However, *Slfn14^fl/fl^* PF4-Cre platelets did not aggregate or secrete ATP in response to 0.03 U/mL thrombin (Figure 4, C, D, and G) and showed a reduced aggregation and ATP secretion at 0.06 U/mL (Figure 4, E, F, and H). This concurs with *SLFN14* patient data in which reduced aggregation and secretion in response to PAR1 and ADP is observed. Normal platelet aggregation and ATP secretion were observed in response to high and low doses of collagen and CRP (Supplemental Figure 2).

### Slfn14^fl/fl^ PF4-Cre mice have fewer in situ MKs in the bone marrow compared with controls.

**Figure 5:**
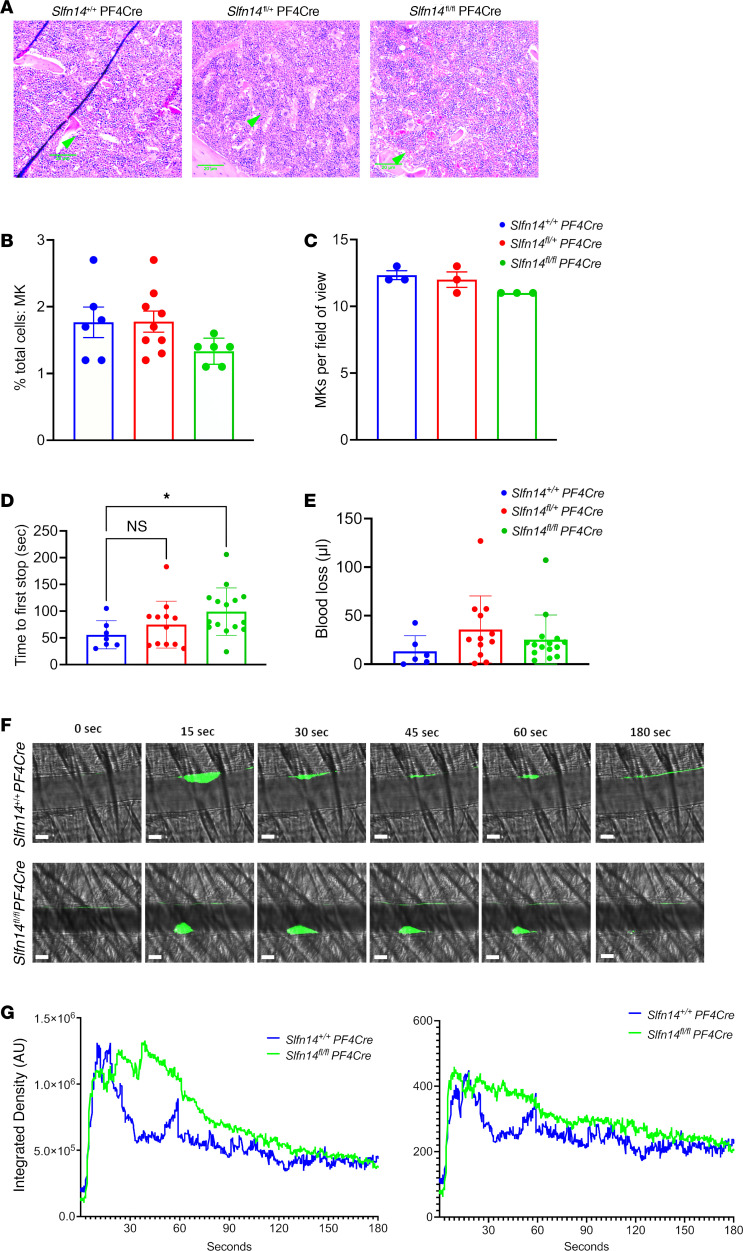
In situ MK assessment, tail bleeding, and laser injury–induced thrombus formation in *Slfn14^fl/fl^* PF4-Cre mice. (**A**) Representative images of H&E-stained femur sections from *Slfn14^+/+^* PF4-Cre, *Slfn14^+/fl^* PF4-Cre, and *Slfn14^fl/fl^* PF4-Cre mice. Femurs were fixed in 4% formaldehyde and decalcified before sectioning, staining, and quantification of MK number per field of view. MKs are indicated by green arrowheads. Scale bars: 20 μm. (**B**) Quantification of MK number per field of view from 3 mice per genotype. Two femurs per mouse were sectioned, and 10 to 13 fields of view per section were quantified blind. One-way ANOVA was used to assess significance. (**C**) Percentage of MKs out of total cells in native BM in *Slfn14^fl/fl^* PF4-Cre and *Slfn14^fl/+^* PF4-Cre mice compared with *Slfn14^+/+^* PF4-Cre controls. (**D**) Tail bleeding time assay where 3 mm of tail was removed, and mice bleeding time until first stop was measured. Data presented are mean time in seconds; each data point represents 1 animal. *n* = 7–15 mice per genotype (*P < 0.05). (**E**) Total blood loss up to 20 minutes following start of experiment. Data are presented in microliters; each data point represents 1 animal. *n* = 7–15 mice per genotype. (**F** and **G**) Laser-induced thrombus formation in *Slfn14* PF4-Cre mice. Mice were injected with DyLight488-conjugated anti-GPIbβ antibody (0.1 μg/g body weight). Arterioles of cremaster muscles were subsequently injured by laser. (**F**) Representative composite brightfield and fluorescence images of platelets (GPIbβ). Scale bar: 10 μm. (**G**) Each curve represents median integrated fluorescence intensity (left panel) in AU for 31–32 injuries in 4 mice per genotype and mean area (right panel) in pixels (Supplemental Videos 1, *Slfn14^+/+^* PF4-Cre, and 2, *Slfn14^fl/fl^* PF4-Cre).

In situ MKs were examined in native femur BM. BM histology revealed MKs within the BM, with normal morphology, including the characteristic polyploid nuclei, across all genotypes (Figure 5A). Slightly fewer MKs were observed in *Slfn14^fl/fl^* PF4-Cre mice; however, this did not reach significance (Figure 5B; *P* = 0.06). We analyzed the proportion of CD41^+^/CD42^+^ cells (MKs) as a percentage of all BM cells levels in flushed whole BM from mouse femurs and tibias by flow cytometry. Live, single cells were selected and MKs identified as double-positive populations determined by antibody staining as shown previously (1). There was a trend for fewer MKs in native BM in *Slfn14^fl/fl^* PF4-Cre mice compared with controls, although this did not reach significance (Figure 5C). MK development was examined by flow cytometry, and ploidy levels in the *Slfn14^fl/fl^* PF4-Cre MKs were consistent with *Slfn14^+/+^* PF4-Cre controls (Supplemental Figure 4).

### Slfn14^fl/fl^ PF4-Cre mice show an increased bleeding tendency compared with controls.

To establish if SLFN14 mice had a bleeding phenotype, 3 mm of tail tip was excised and time to bleeding cessation was measured in prewarmed (37°C) saline. Significantly increased bleeding time (time to first stop) was observed in *Slfn14^fl/fl^* PF4-Cre compared with *Slfn14^+/+^* PF4-Cre mice (*P* < 0.05), indicating that SLFN14 downregulation in mice impacts hemostasis in this model (Figure 5, D and E).

### Reduced laser injury–induced thrombus formation in Slfn14^fl/fl^ PF4-Cre mice.

To assess in vivo thrombus formation, *Slfn14^fl/fl^* PF4-Cre and control *Slfn14^+/+^* PF4-Cre mice were injected with fluorescently conjugated anti-GPIbβ antibody to label platelets prior to induction of laser injuries to the arterioles of the cremaster muscles. Intravital analysis showed that thrombi developed at different kinetics in *Slfn14^fl/fl^* PF4-Cre and *Slfn14^+/+^* PF4-Cre control mice. *Slfn14^fl/fl^* PF4-Cre mice formed delayed thrombi, which increased progressively, but platelets embolized continuously, delaying the time to reach thrombi peak size compared with *Slfn14^+/+^* PF4-Cre control mice (Figure 5, F and G, and Supplemental Videos 1 and 2). Therefore, the defect in platelet SLFN14 resulted in delayed thrombus formation, explaining the bleeding phenotype in patients.

### RNA-Seq shows genes enhanced in translation and transcription pathways in MKs and platelets from the Slfn14^fl/fl^ PF4-Cre mouse model.

**Figure 6:**
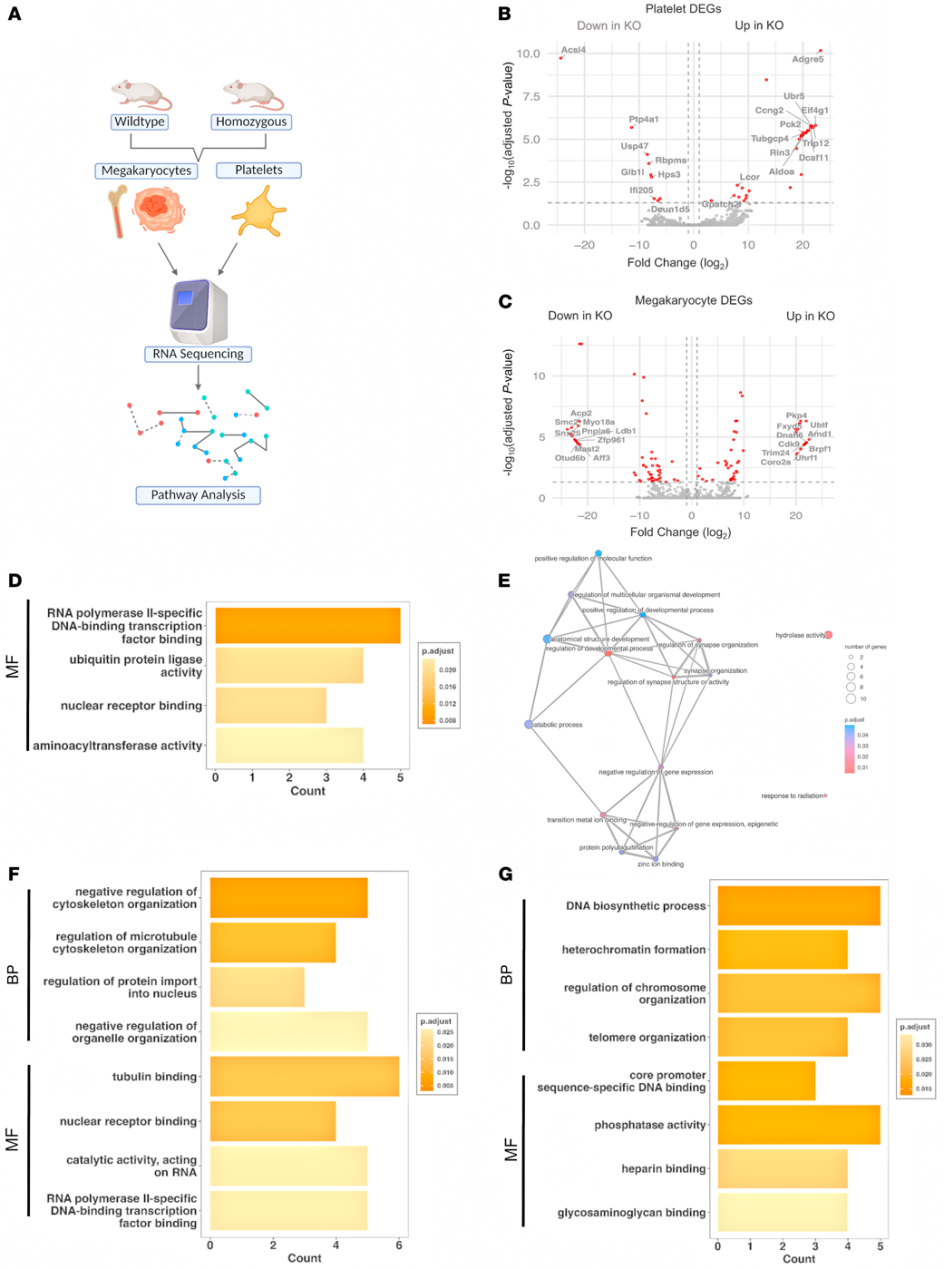
RNA-Seq, GO, and pathway analysis of *Slfn14^fl/fl^* PF4-Cre versus *Slfn14^+/+^* PF4-Cre control mice. (**A**) RNA-Seq analysis and workflow from murine samples. (**B** and **C**) Volcano plots of RNA-Seq target genes significantly up- or downregulated from (**B**) platelets and (**C**) MKs derived from *Slfn14^fl/fl^* PF4-Cre mutant and *Slfn14^+/+^* PF4-Cre control mice. The top 10 up- and downregulated protein coding transcripts are annotated. (**D**) GO enrichment analysis of upregulated genes in *Slfn14^fl/fl^* PF4-Cre mutant platelets. Selection of upregulated subontology term “molecular function” (MF). Significantly enriched GO terms were selected based on adjusted *P* value < 0.05. (**E**) GSEA map based on DEGs in KO platelets. (**F**) GO enrichment analysis of upregulated genes in *Slfn14^fl/fl^* PF4-Cre mutant MKs. Selection of upregulated subontology terms “biological process” (BP) and MF. (**G**) GO enrichment analysis of downregulated genes in *Slfn14^fl/fl^* PF4-Cre mutant MKs. Selection of downregulated subontology terms BP and MF.

To identify the mechanism through which SLFN14 may regulate megakaryopoiesis and platelet function, we performed RNA-Seq analysis to define alterations in gene activity arising as a result of conditionally knocking down *Slfn14* in vivo. Bulk RNA-Seq was performed on total RNA extracted from mature murine *Slfn14^fl/fl^* PF4-Cre–derived MKs and peripheral blood platelets and compared with *Slfn14^+/+^* PF4-Cre control MKs/platelets (Figure 6A). All individual samples were run in triplicate and normalized before comparisons between each *SLFN14* mutant and the WT group. The RNA-Seq gene expression analysis of *Slfn14^fl/fl^* PF4-Cre MKs and platelets identified a number of up- and downregulated genes (Figure 6, B and C). RNA-Seq analysis of *Slfn14^fl/fl^* PF4-Cre platelets identified 26,148 transcripts, with 26 genes found to be significantly upregulated (including *Adgre5*, *Ubr5*, *Ccng2*, and *Tubgcp4*) and 8 genes significantly downregulated (including *Ptp4a1*, *Usp47*, and *Rbpms*) in homozygous mice versus controls (Figure 6B and Supplemental Data 1). This potentially indicates that SLFN14 defects may influence gene expression and regulation in platelets. Similarly, we investigated the role of SLFN14 in megakaryopoiesis, and RNA-Seq analysis of *Slfn14^fl/fl^* PF4-Cre MKs identified 42,317 transcripts, with 45 genes found to be significantly upregulated (including *Pkp4*, *Fxyd5*, *Ubtf*, and *Cdk9*) and 52 genes significantly downregulated (including *Acp2*, *Smc2*, *Myo18a*, and *Snx25*) in homozygous mice versus controls (Figure 6C and Supplemental Data 1).

To compare our transcriptomic dataset from a previous human study (10), we performed a gene-level overlap analysis between the DEGs in mouse platelets and MKs and those identified in platelets from patients carrying the heterozygous SLFN14 K219N variant (10). We found that 30 of 92 DEGs (~33%) in our MK dataset overlap with the SLFN14 K219N patient platelet DEGs and 10 of 33 DEGs (~30%) in our platelet dataset also overlap (Supplemental Figure 5).

To extend findings from SLFN14 K219N patient platelets reported previously (10), we examined ribosomal protein S6 levels in SLFN14-deficient mouse platelets and observed a clear increase, consistent with enhanced mTORC1 signaling (Supplemental Figure 6A). Supporting this observation, increased S6 levels were also detected in platelets from a patient carrying the SLFN14 K219N variant (Supplemental Figure 6B). These data highlight SLFN14 as a critical regulator of ribosome homeostasis.

### Pathway and gene enrichment analysis in the Slfn14^fl/fl^ PF4-Cre mouse model.

Following initial analysis, the differentially regulated genes were submitted to Gene Ontology (GO) analysis for biological processes and molecular functions (Figure 6, D, F, and G). In *Slfn14^fl/fl^* PF4-Cre platelets, the upregulated genes were predominantly enriched for the terms RNA polymerase II–specific DNA-binding transcription factor binding, ubiquitin protein ligase activity, nuclear receptor binding, and aminoacyltransferase activity (Figure 6D). Gene set enrichment analysis (GSEA) was performed on platelet DEGs and visualized in an enrichment map, highlighting pathways related to molecular function regulation, developmental processes, and stress response mechanisms (Figure 6E).

In *Slfn14^fl/fl^* PF4-Cre MKs, the upregulated genes revealed enrichment in the regulation of cytoskeleton organization, protein import into the nucleus, tubulin binding, nuclear receptor binding, and RNA polymerase II–specific DNA-binding transcription factor binding (Figure 6F). Conversely, GO analysis for downregulated genes in *Slfn14^fl/fl^* PF4-Cre MKs demonstrated enrichment in terms such as DNA biosynthetic process, heterochromatin formation, regulation of chromosome organization, core promoter sequence-specific DNA binding, and phosphatase activity (Figure 6G). These findings suggest that, in control cells, the presence of SLFN14 inhibits processes linked to cytoskeletal and nuclear regulation, while promoting others, including chromosomal maintenance.

## Discussion

SLFN14 is a hematopoiesis-specific endoribonuclease with an unidentified role. *SLFN14* missense mutations have previously been found in 8 unrelated families worldwide with inherited thrombocytopenia and platelet function defects (3). Human patients with mutations in *SLFN14* (K219N, V220D, and K218E) all presented with mild macrothrombocytopenia and reduced aggregation and ATP secretion responses to collagen, ADP, and PAR1 receptor activating peptide. However, it remained unclear how these *SLFN14* mutations contribute to platelet function defects in humans. To elucidate the platelet activation and signaling pathways further, we recalled and studied 2 of the 3 *SLFN14*-mutated families (V220D and K219N) reported in the original study (3). In this study, *SLFN14* K219N– and V220D–mutated patients showed reduced phosphorylation of Syk-Y525/526 and LAT-Y200 in response to collagen and CRP as well as reduced phosphorylation of ERK1/2-Thr202/204 and AKT-Tyr473 downstream of ADP and PAR1 peptide. Therefore, *SLFN14* mutations in humans perturb platelet signaling downstream of GPVI (Figure 1).

To investigate the function of SLFN14 further, we generated a new murine model utilizing Cre recombinase under the PF4 promoter to efficiently ablate *Slfn14* in MKs and platelets (11). In this study, we investigated both *Slfn14* PF4-Cre heterozygous (*Slfn14^fl/+^* PF4-Cre) and homozygous (*Slfn14^fl/fl^* PF4-Cre) mice alongside WT controls (*Slfn14^+/+^*). *Slfn14^fl/+^* PF4-Cre mice pairings bred with normal Mendelian inheritance ratios, suggesting no embryonic lethality, as observed in our previously published *Slfn14-K208N* model. PF4-Cre–mediated deletion of exons 2 and 3 of *Slfn14* resulted in loss of *SLFN14* gene expression using quantitative RT-PCR. *Slfn14* mRNA expression was reduced in both heterozygous and homozygous mice compared with WT controls in both platelet and MK-derived RNA samples. Hematological analyses revealed that *Slfn14^fl/fl^* PF4-Cre mice display a mild macrothrombocytopenia phenotype and normal levels of platelet glycoprotein receptors. Despite normal receptor expression and platelet structure, reduced platelet function and fewer in situ MKs suggest that *SLFN14* mutations may only affect platelet functionality and downstream signaling rather than their production in the BM.

Overall flow cytometry (FSC and SSC) analysis and TEM of *SLFN14^fl/fl^* PF4-Cre and *Slfn14^fl/+^* PF4-Cre mice showed a slight increase in platelet area compared with WT and heterozygous littermates, indicating altered platelet morphology. However, no differences in δ granule levels were observed in the *Slfn14^fl/fl^* PF4-Cre mice. Additionally, a significant increase was observed in the *Slfn14^fl/+^* PF4-Cre mice for α granules but not in *Slfn14^fl/fl^* PF4-Cre mice, which was confirmed by SSC analysis. Platelet activation in response to major agonists was assessed by flow cytometry and overall showed *Slfn14^fl/+^* PF4-Cre and *Slfn14^fl/fl^* PF4-Cre platelets to have normal responses to major platelet agonists.

*SLFN14* patients were originally recruited to the GAPP study based on their bleeding tendency. Here, in our mouse model using tail bleeding studies we show an increased bleeding tendency in *Slfn14^fl/fl^* PF4-Cre mice, indicating that SLFN14 downregulation impacts hemostasis in murine SLFN14, which is in keeping with the human SLFN14-associated bleeding phenotype.

In vivo studies via intravital microscopy found that delayed thrombus formation was observed in *Slfn14^fl/fl^* PF4-Cre mice compared with littermate controls. Thrombi in *Slfn14^fl/fl^* PF4-Cre mice initially formed at the same rate as control mice, but generally there was a weaker response overall where thrombi persisted over a longer time frame. These defects in the formation and stability of in vivo thrombi are likely attributable to the abnormal platelet dysfunction in these mice. Indeed, our previous study showed reduced thrombus formation and stability in *Slfn14*^K208N/+^ mice (1). Our data show that the thrombus instability could explain why the SLFN14 patients display an excessive bleeding phenotype.

RNA-Seq analysis of *Slfn14^fl/fl^* PF4-Cre platelets identified 26,148 transcripts, with 26 genes found to be significantly upregulated and 8 genes significantly downregulated in *Slfn14^fl/fl^* PF4-Cre compared with the controls. Many of these genes play critical roles in processes such as signaling, metabolism, cytoskeleton organization, and gene expression regulation, which are necessary for platelet production, function, and maintenance. Dysregulation in these genes can lead to irregularities in platelet function, potentially causing bleeding disorders. Signal transduction, ion transport, phosphorylation, protein transport, and GPCR signaling pathways showed enrichment, indicating their functional role in *Slfn14^fl/fl^* PF4-Cre mice and that they are important and directly involved in platelet activation, signaling, and functions. Furthermore, key biological processes vital for MK development and platelet production are enriched, including regulation of transcription, cell differentiation, positive regulation of cell proliferation, and apoptosis processes. Additionally, GSEA, which predicts molecular interactions, identified a number of crucial pathways in platelet activation and function. Regulation of actin cytoskeleton, calcium signaling, JAK-stat signaling, phospholipase C signaling, and RAP1 signaling are directly involved in platelet activation and function, with metabolic pathways and ERK1/2 signaling essential for MK development and platelet production. For example, Septin 2, involved in the cell cytoskeleton, has been identified in human platelets (14), and in this study, we observed that Septin 2 was significantly upregulated in platelet samples and interestingly downregulated in MKs.

Furthermore, comparison of RNA-Seq data in *Slfn14^fl/fl^* PF4-Cre mice with littermate controls suggested that GPCR signaling was the most enriched pathway in biological processes. In addition, when looking at the molecular function enrichment, GPCR activity was significantly enriched, being present in the top 3 hits. This enrichment analysis highlights what was observed in platelet function assays within this study in which there was a dysfunction in *Slfn14^fl/fl^* PF4-Cre platelets in response to thrombin. This could potentially be explained by the reduced signaling in the Gq pathway triggered by PAR receptors and P2Y_1_ and P2Y_12_ receptors for ADP. This is parallel with *SLFN14* patient data that show reduced aggregation and secretion in response to PAR1 and ADP (3). It is well known that GPCRs can influence platelet function by mediating the response to various agonists, including ADP, thromboxane, and thrombin (15).

Comparisons between our mouse transcriptomics dataset and a previous human study (10) revealed that around one-third of the transcriptional changes observed in our model occur in a completely independent dataset, across species and genetic backgrounds. However, it is important to note that there are key differences between the model systems: Ver Donck et al. studied platelets from patients with a heterozygous SLFN14 K219N variant, which expresses a mutant protein with potential dominant-negative or gain-of-function properties (10). In contrast, our model used platelet-specific KO mice, where *Slfn14* was completely absent in the MK/platelet lineage. Therefore, to our knowledge, our study represents the first in vivo model of complete *Slfn14* loss of function in platelets, enabling a direct assessment of its essential role without confounding effects from mutant protein expression or the presence of a remaining WT allele.

The previous study also found alterations in the rRNA processing and mTORC1 signaling axis in SLFN14 K219N models, which could be an important mechanism contributing to the phenotype (10). Therefore, to address this, we specifically examined ribosomal protein S6 levels and found them to be increased in both mouse SLFN14-deficient and human SLFN14-mutant platelets. As S6 is a key downstream target of mTORC1 signaling, this observation supports the notion of enhanced mTORC1 activity and conserved translational stress responses across species.

In summary, to investigate the functional role of SLFN14 and its mutations in platelets, we have characterized a new *Slfn14* PF4-Cre mouse model with particular focus on platelet function and expression. Detailed activation and signaling studies of platelets from the originally reported patients with SLFN14 mutations (V220D and K219N) showed reduced ITAM and GPCR signaling, all of which are downstream of GPVI. Furthermore, to define the role of SLFN14 in MKs and platelet function, we generated a new murine model with a platelet- and MK-specific *Slfn14* deletion and employed extensive hematological analysis, intravital microscopy, and RNA-Seq in platelets and MKs from these mice. *Slfn14^fl/fl^* PF4-Cre mice presented with macrothrombocytopenia and reduced platelet function in response to thrombin-associated agonists and therefore closely resembled the human phenotype. Extensive platelet and MK phenotyping, in addition to GO analysis, indicated that SLFN14 contributes to pathways related to DNA synthesis, chromatin structure, chromosome organization, and transcription factor binding; therefore, mutations within *SLFN14* in both humans and mice disrupt platelet production and function in both species. In conclusion, we have investigated how SLFN14 deletion can lead to the bleeding phenotype in mice as with patients with *SLFN14* mutations.

## Methods

### Sex as a biological variable.

Both males and females were used in this study, with the exception of male mice only for the cremaster model of thrombosis.

### Patient testing.

Platelet counts, MPV, and other hematological parameters were obtained on a Sysmex XN1000 full blood analyzer (Sysmex UK). Blood was drawn from the patients with the V220D and K219N *SLFN14* mutations on 2 separate occasions, and a same-day healthy donor control was collected alongside.

### Mice.

*SLFN14* conditional KO mice were generated by crossing PF4-Cre^+^ transgenic mice with SLFN14-floxed mice generated using the Cre/LoxP system. *Slfn14^+/+^* PF4-Cre were used as controls and compared with *Slfn14^fl/+^* PF4-Cre and *Slfn14^fl/fl^* PF4-Cre. Mice were genotyped in house using PCR and custom primers to detect the floxed allele. PF4-Cre recombinase was detected in a separate PCR. All primer sequences are shown in supplemental material.

### Platelet preparation.

Blood was collected into 1:10 (v/v) acid-citrate-dextrose anticoagulant from the inferior vena cava of mice under terminal anesthesia (isoflurane/O_2_ 5% gas). Washed platelets were prepared as previously described (1).

### TEM.

Platelets were prepared as previously described (1) and subsequently fixed in 2.5% glutaraldehyde solution in phosphate buffer, embedded in 100% resin, and sectioned at 0.8 μm.

### Platelet function assays.

Platelet function was assessed by flow cytometry, light transmission aggregometry, and spreading as previously described (1). Platelet activation and signaling of major platelet receptors GPVI, PAR1, P2Y_1_, and P2Y_12_ were assessed by Western blot and as previously described (16). Antibodies and platelet agonists are listed in Supplemental Tables 1 and 2.

### Isolation of native MKs for RNA-Seq.

BM was isolated from tibias and femurs by centrifugation (2,500*g*, 40 seconds), and MKs were isolated using CD61-conjugated magnetic beads (Miltenyi Biotec). MK RNA was extracted using a low-input combination method with TRIzol reagent (Invitrogen) and an RNeasy MinElute Kit (Qiagen) following the manufacturer’s protocol.

### Tail bleeding assay.

All experiments were double-blind and conducted on 20–29 g *Slfn14^fl/fl^* PF4-Cre, *Slfn14^fl/+^* PF4-Cre, and *Slfn14^+/+^* PF4-Cre mice. Mice were anesthetized by intraperitoneal administration of ketamine/medetomidine, and 3 mm of tail tip was excised using a sterile razor blade. Mice were bled into prewarmed saline (37°C). Time until first cessation of bleeding was recorded in seconds up to 20 minutes, and total blood loss was measured.

Following tail bleeding, blood was centrifuged at 700*g* for 5 minutes and the pellet resuspended in deionized water to lyse RBCs. Hemoglobin release was measured by absorbance at 405 nm using a microplate reader. To quantify blood loss, absorbance values were interpolated against a standard curve prepared from serial dilutions of freshly drawn mouse blood lysed in water under identical conditions.

### In vivo thrombosis assay.

Laser-induced injury of cremaster arterioles was performed and analyzed as previously described (1, 17).

### Platelet and MK RNA extraction for RNA-Seq.

Platelets and MKs were isolated as previously described (1), and RNA was extracted using the RNeasy mini kit (Qiagen) according to the manufacturer’s instructions.

### RNA-Seq.

RNA-Seq was performed on platelet and MK RNA in triplicate groups from individual mice (*Slfn14^fl/fl^* PF4-Cre, *Slfn14^fl/+^* PF4-Cre, and *Slfn14^+/+^* PF4-Cre) by Source Bioscience UK Limited. RNA samples were quantified via a fluorometric method involving an Invitrogen Qubit RNA assay and qualified using the Agilent Bioanalyzer 2100 (Supplemental Figure 7). RNA integrity number cutoff was > 7 to be used for sequencing. The library preparation method is detailed in Supplemental Methods.

### Gene enrichment and pathway analysis.

Sequencing analysis was conducted with R. Differential gene expression analysis was performed using the DESeq2 package (18). Ensembl and biomaRt were used to map Ensembl gene identifiers and retrieve associated gene annotations from the Ensembl database (19, 20).

Functional enrichment analysis was performed using the clusterProfiler package in R, with mouse gene annotations retrieved from org.Mm.eg.db and supported by the AnnotationDbi framework. GO terms were obtained from the GO resource. DEGs and enrichment results were visualized using the enrichplot and ggplot2 packages (21–23).

### Statistics.

Data are presented as mean ± SEM unless otherwise stated. *P* < 0.05 was considered significant. All analyses were conducted using GraphPad Prism software v9.3.

### Study approval.

Consented patients were recruited to the GAPP study as previously described (3, 5) and approved by the United Kingdom National Research Ethics Service by the Research Ethics Committee of West Midlands (06/MRE07/36). Blood samples were obtained in accordance with the Declaration of Helsinki. All animals were bred on C57BL/6J background in accordance with United Kingdom Home Office regulations (PPLs P53D52513 and PP3749922) and the Animals (Scientific Procedures) Act 1986 with the approval of the local ethics committee at the University of Birmingham.

### Data availability.

RNA-Seq data are available in the National Center for Biotechnology Information Sequence Read Archive (Accession number PRJNA1301939) BioSample public repository under accession numbers SAMN50221041 and SAMN50221042. Values for reported data are provided in the Supporting Data Values file.

## Author contributions

NVM designed the study, recruited the patients, and undertook governance of the study. RJS, XS, GHMA, ME, LGQ, SRMS, AA, EJH, NK, AB, AVP, NSP, JR, DK, SGT, SJM, ZN, and NVM extracted or generated clinical or experimental data and interpreted the results. RJS, XS, and NVM wrote the manuscript. All authors read and approved the final version of the manuscript.

## Supplementary Material

Supplemental data

Supplemental data set 1

Unedited blot and gel images

Supplemental video 1

Supplemental video 2

Supporting data values

## Version 1 08/12/2025

In-Press Preview

## Version 2 10/15/2025

Electronic publication
