# Supplementary material for: Platelet-specific SLFN14 deletion causes macrothrombocytopenia and platelet dysfunction through dysregulated megakaryocyte and platelet gene expression
Source: J Clin Invest. 2025 Aug 12;135(20):e189100. doi: 10.1172/JCI189100 (PMC12520693; doi:10.1172/JCI189100)

Supplemental Files - *Stapley et al. 2025*

***Slfn14-PF4Cre* mice genotyping primer sequences**

SLFN14 forward: 5' – GGCTCAGTTGGTAGCTAGAG – 3’

SLFN14 reverse: 5' – CAGACATGACCTCATGGAAC – 3’

PF4Cre forward: 5'- CCCATACAGCACACCTTTTG – 3’

PF4Cre reverse: 5'- TGCACAGTCAGCAGGTT – 3’

Quantitative RT-PCR *Slfn14* mRNA gene expression

Forward Primer: 5' – AGCCCTTCTGTTGTGTCGTGT – 3’

Reverse Primer: 5' - GCCTGAAGAAGGATCTGACTGA– 3’

Platelet bulk RNA sequencing

Libraries were prepared using the NEBNext® Single Cell/Low Input RNA Library Prep Kit for Illumina® according to the manufacturer’s protocol. During this process, the libraires were indexed using NEBNext® Multiplex Oligos for Illumina® (96 Unique Dual Index Primer Pairs Set 4). The prepared libraries were quantified via a fluorometric method involving an Invitrogen Qubit dsDNA assay and qualified using electrophoretic separation on the Agilent BioAnalyzer 2100.

MK bulk RNA sequencing

Libraries were prepared using the NEBNext® Ultra™ II Directional RNA Library Prep Kit for Illumina with NEBNext® rRNA Depletion Kit v2 (Human/Mouse/Rat) according to the manufacturer’s protocol. During this process, the libraires were indexed using NEBNext® Multiplex Oligos for Illumina® (96 Unique Dual Index Primer Pairs) Set 4. The prepared

libraries were quantified via a fluorometric method involving a Promega QuantiFluor dsDNA assay and qualified using electrophoretic separation on the Agilent TapeStation 4200.

Supplemental Table 1: **Antibodies used in flow cytometry**

**Antibody**	**Host species, conjugate, catalogue number**	**Manufacturer**	**Dilution**
CD41 (αIIb) – mouse	Rat, APC, 133913	Biolegend	FCC 1:200<br>FC 1:100
CLEC2 - mouse	Rat, FITC, 17D9MCA5700F	Bio-Rad Laboratories	FC 1:100
CD42a (GpIb) – mouse	Rat, FITC, M040-1	Emfret Analytics	FC 1:100
CD49b (α2) – mouse	Rat, FITC, M071-1	Emfret Analytics	FC 1:100
CD41/CD61 (integrin αIIbβ3) – mouse	Rat, FITC, M025-1	Emfret Analytics	FC 1:100
GPVI – mouse	Rat, FITC, M011-1	Emfret Analytics	FC 1:100
CD62P (P-selectin) – mouse	Rat, FITC, M130-1	Emfret Analytics	FC 1:100
CD41/CD61 activated (JON/A) – mouse	Rat, PE, M023-2	Emfret Analytics	FC 1:100
**Isotype controls**			
IgG	Rat, FITC, P190-1	Emfret Analytics	FC 1:100
IgG2a	Rat, PE, 400507	Biolegend	FC 1:100
IgG1	Rat, APC, 400411	Biolegend	FC 1:100
IgG2b	Rat, FITC, MCA6006F	Bio-Rad Laboratories	FC 1:100
IgG2b	Rat, APC e-780, 15321650	eBioscience	FC 1:100

<u>Supplemental Table 2:</u> Platelet agonists

**Agonist**	**Receptor interaction**	**Manufacturer, catalogue number**
**ADP**	P2Y1, P2Y12	Sigma, A2754
**Collagen**	GPVI, αIIbβ3	Takeda, 1130630
**CRP (collagen related peptide)**	GPVI	Provided by Prof. Farndale; cross-linked in-house
**PAR4-peptide**	PAR4	Alta biosciences
**Thrombin**	PAR1, PAR4 (PAR3 and PAR4 in mice)	Sigma, T4648
**U46619**	TP (thromboxane A_2)	Sigma, D8174

Supplementary figure 1: Chi square analyses to assess for Mendelian inheritance patterns shows both (**i**) flox and (**ii**) PF4Cre alleles were inherited within Mendelian ratios. Data are from 21 litters of heterozygote pairings, average litter size=6 mice.

i

Slfn14$^{fl/+}$ X Slfn14$^{fl/+}$	Expected	Observed
Total	105	104
SLFN14$^{+/+}$	26	24
SLFN14$^{fl/+}$	53	49
SLFN14$^{fl/fl}$	26	31
Chi Square GOF		1.417
DF		2
P-value	ns	0.526

ii

PF4Cre + X PF4Cre -	Expected	Observed
Total	106	104
PF4Cre +	53	59
PF4Cre -	52	45
Binomial Test		
P-value	ns	0.202

Supplementary figure 2: *Slfn14-PF4Cre* platelets show normal aggregation and secretion in response to both high and low doses of collagen and CRP. All assays were performed once on platelet samples from all mouse genotypes.

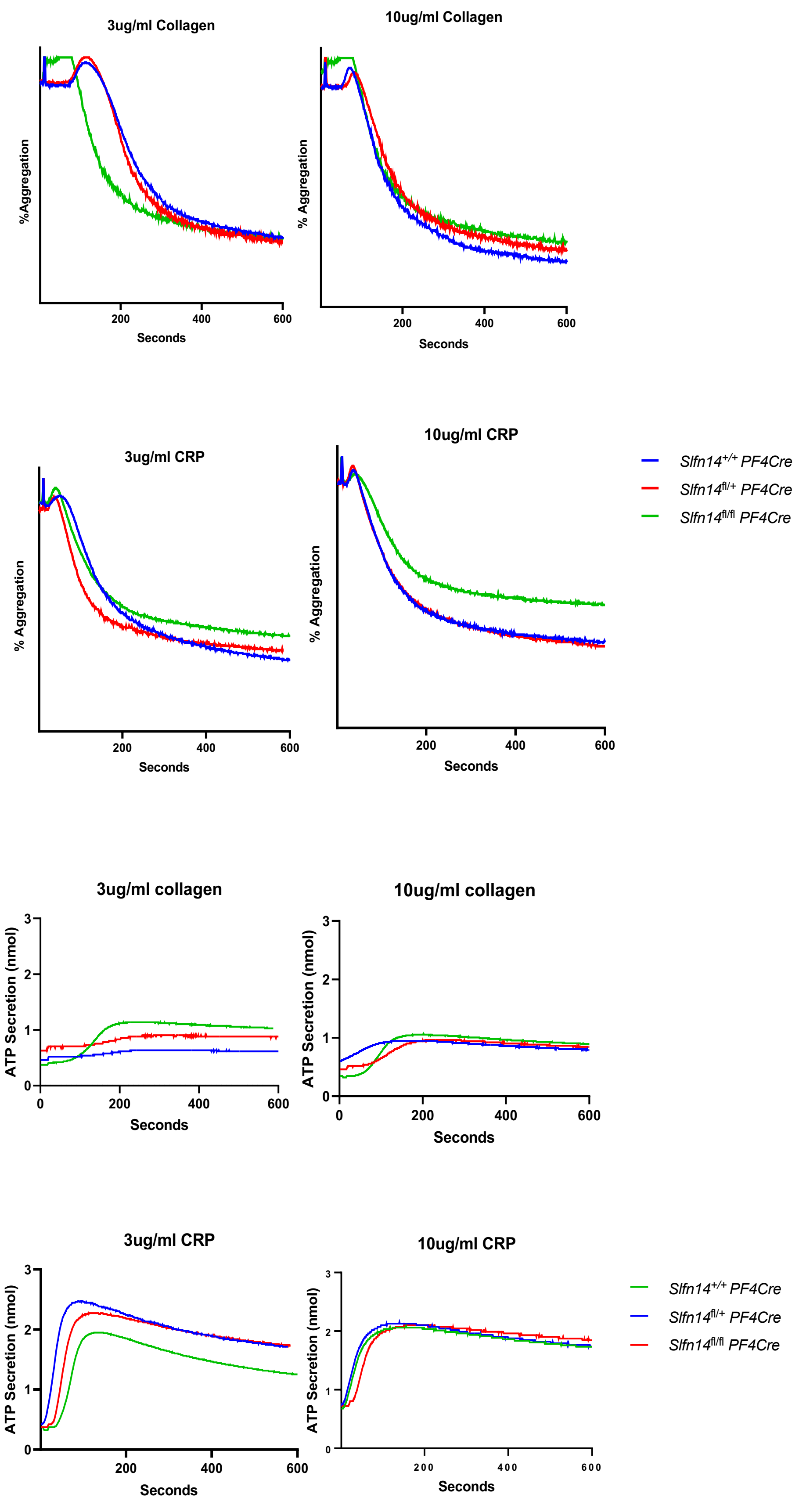

Supplementary figure 3: *Slfn14*-PF4Cre platelets demonstrate no significant difference in spreading on collagen and fibrinogen in comparison to control platelets. (n=1. 30 – 50 platelets analysed per mouse genotype/control.

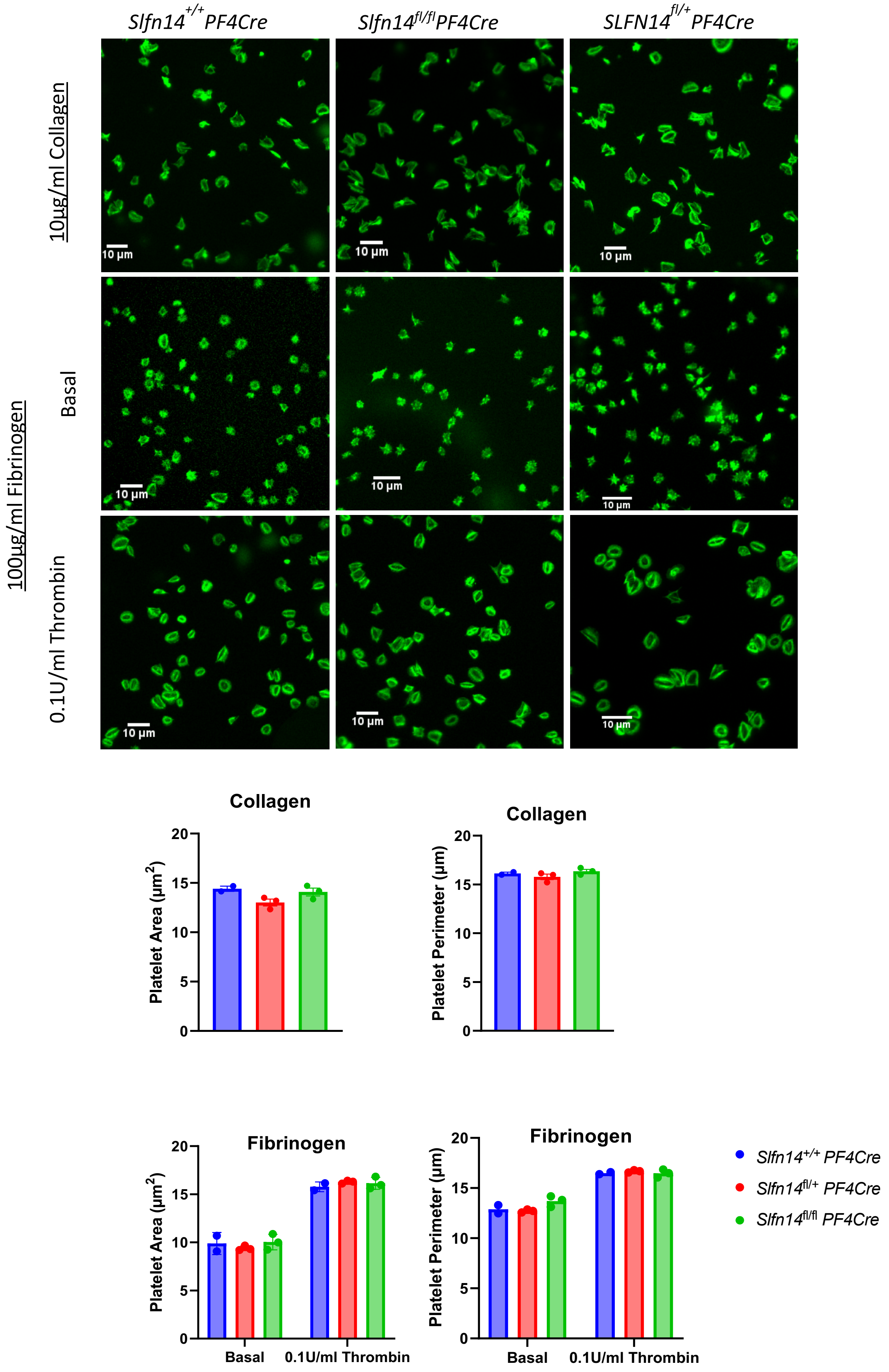

Supplementary figure 4: *Slfn14*$^{fl/fl}$PF4Cre *and Slfn14*$^{fl/+}$PF4Cre MKs display ploidy consistent with control mice.

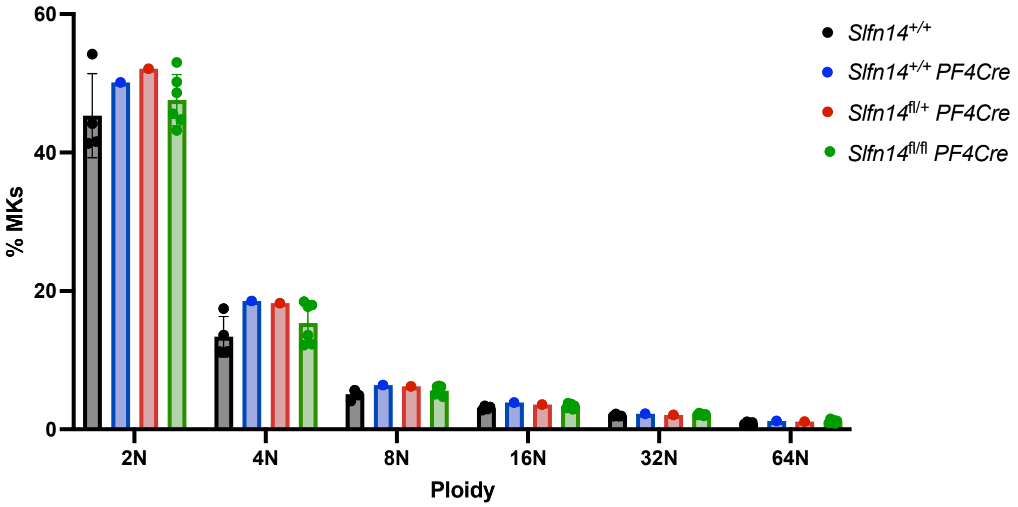

Supplemental figure 5. Venn diagrams showing the overlap between differentially expressed genes (DEGs) in *Slfn14*-deficient mouse model and those reported in *SLFN14* K219N patient platelets (Ver Donck et al., Blood 2023). (A) Mouse megakaryocyte DEGs, (B) mouse platelet DEGs.

A

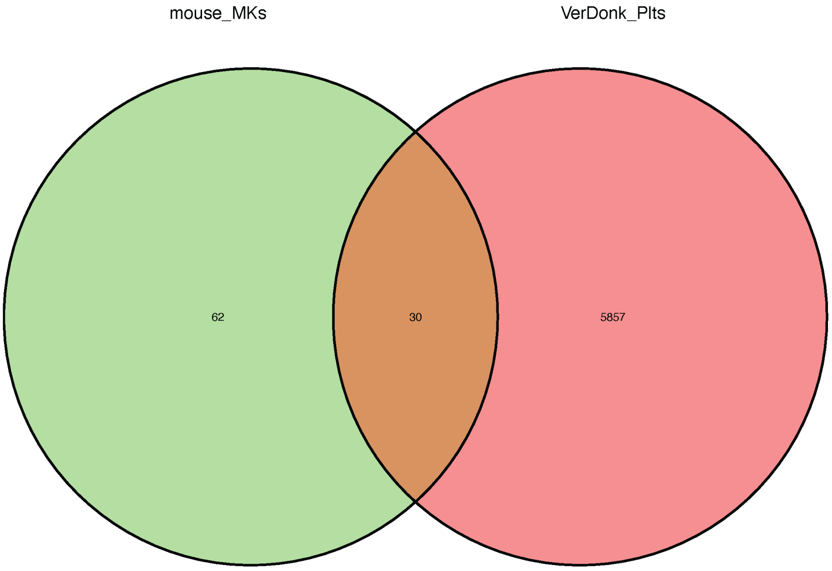

B

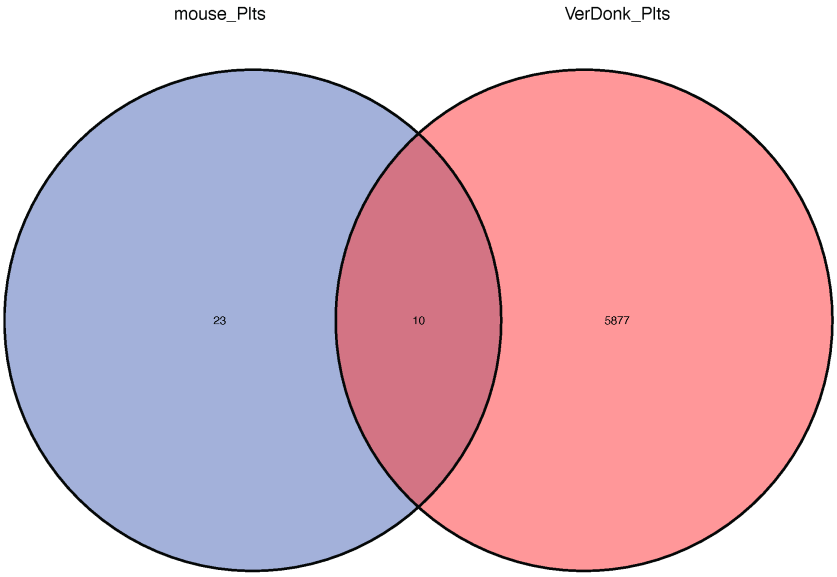

Supplemental Figure 6 A–B. Increased ribosomal protein S6 levels in *Slfn14*-deficient mouse and *SLFN14* mutant human platelets. (A) Western blot analysis of total S6 protein levels in platelets from *Slfn14*$^{+/+}$*;PF4-Cre* and *Slfn14*$^{fl/fl}$*;PF4-Cre* mice. (B) Western blot showing S6 protein levels in platelets from healthy controls (Con) and two *SLFN14* V220D variant patients. *GAPDH* served as a loading control.

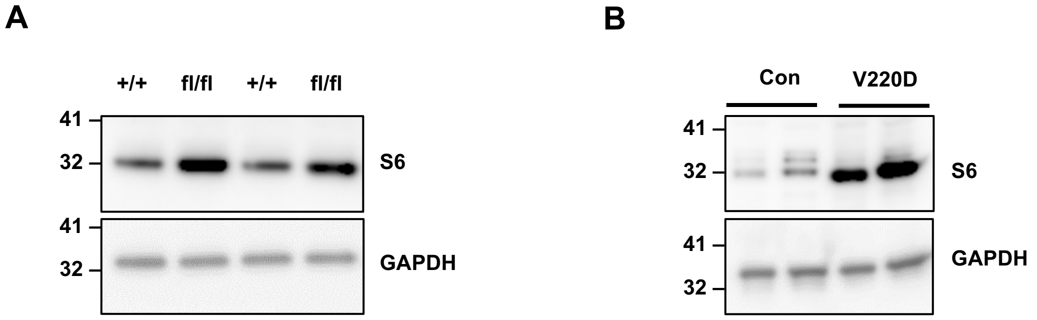

Supplemental figure 7. TapeStation analysis and RNA Integrity values (RIN) of total RNA from all 18 samples derived from *Slfn14*-deficient mouse platelets and megakaryocytes. High Sensitivity RNA ScreenTape® images for RNA derived from *Slfn14*-deficient mouse platelets and megakaryocytes and subsequently submitted for RNAseq analysis.

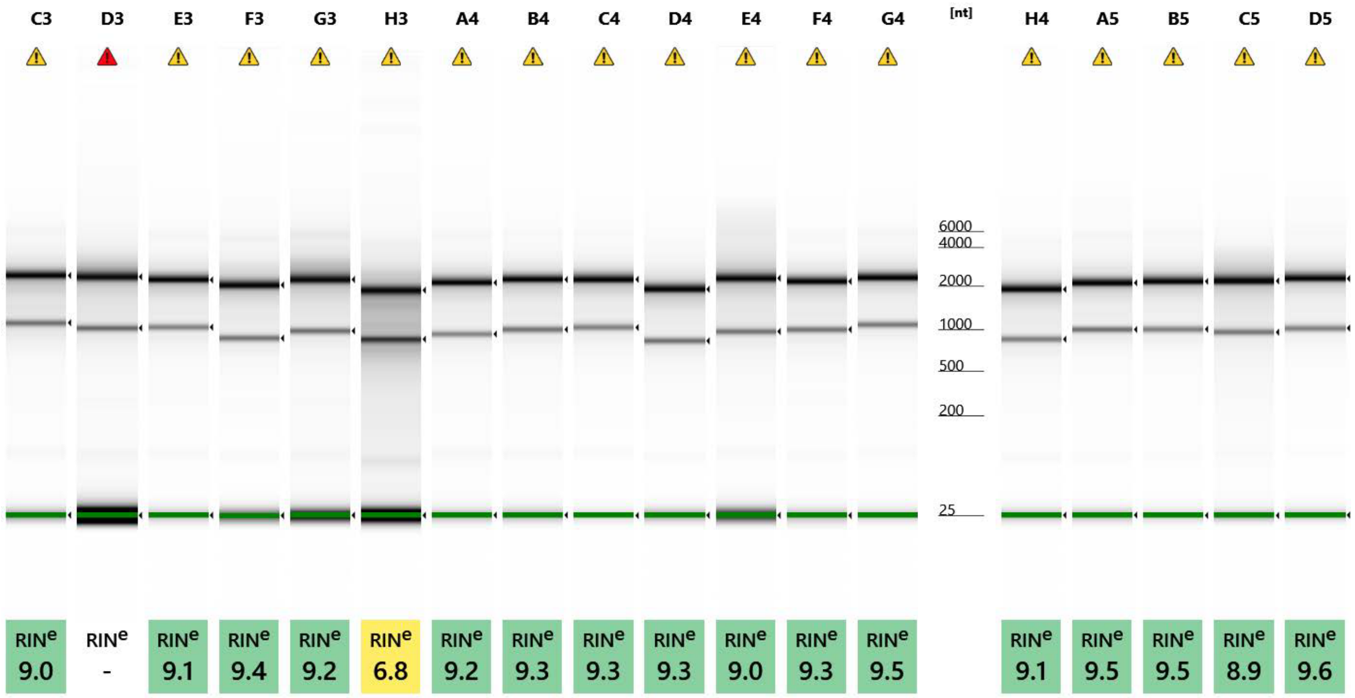

Supplement: Supplemental data [file jci-135-189100-s049.pdf]
