# Supplementary material for: Platelet-specific SLFN14 deletion causes macrothrombocytopenia and platelet dysfunction through dysregulated megakaryocyte and platelet gene expression
Source: J Clin Invest. 2025 Aug 12;135(20):e189100. doi: 10.1172/JCI189100 (PMC12520693; doi:10.1172/JCI189100)

Pan Syk Figure 1

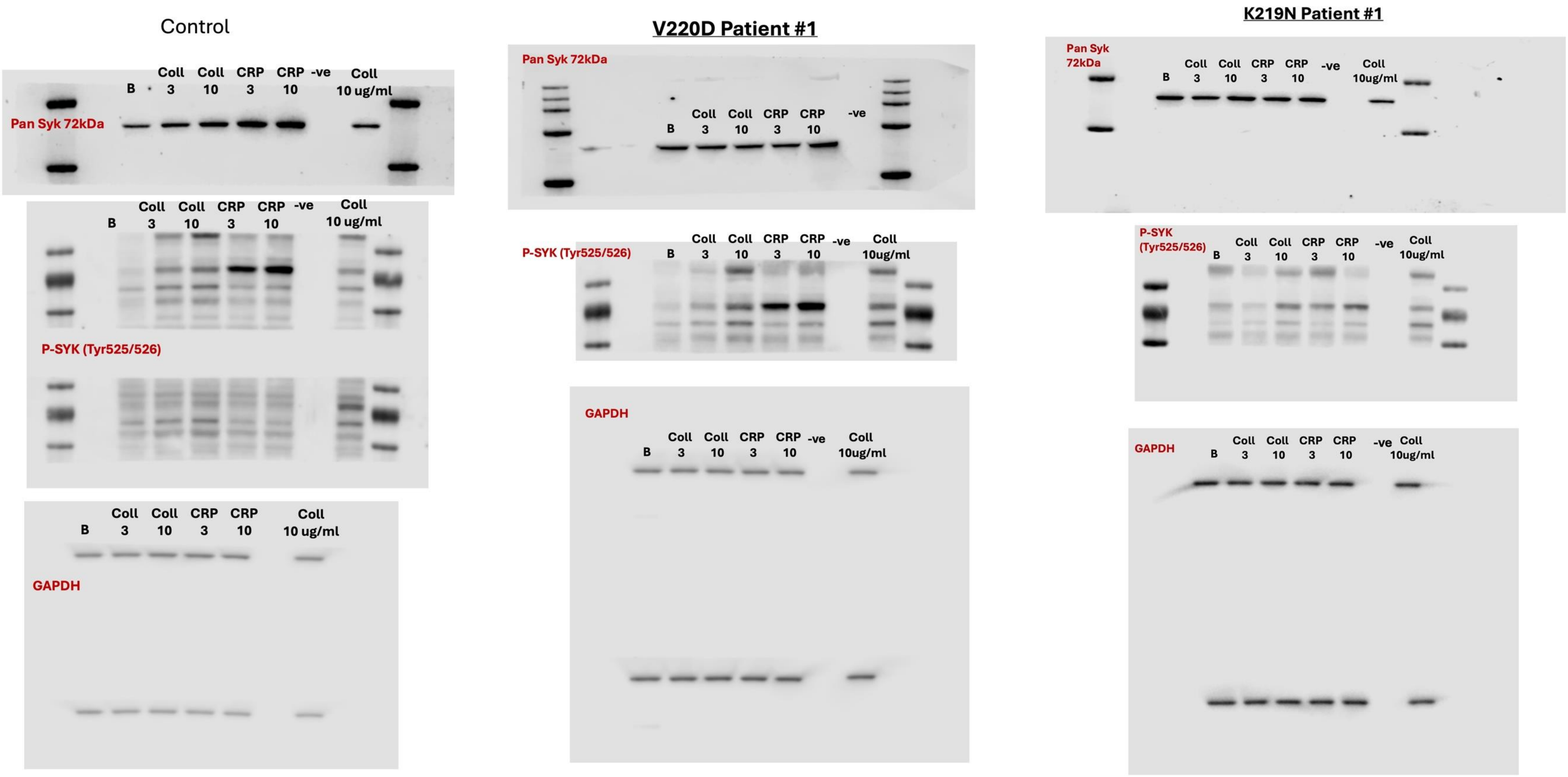

Figure 1b

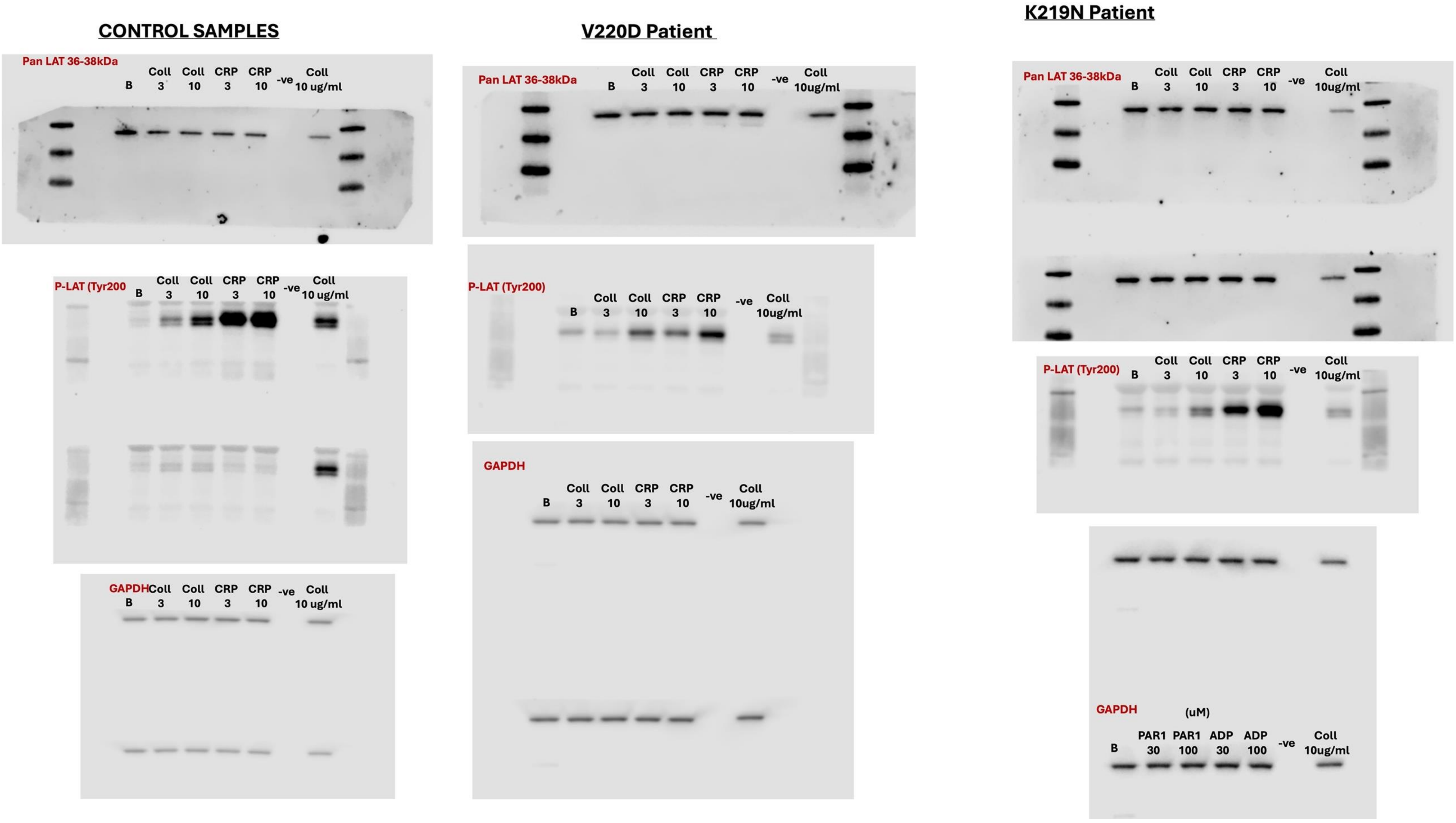
CONTROL SAMPLES
Pan LAT 36-38kDa
B Coll 3 Coll 10 CRP 3 CRP 10 -ve Coll 10 ug/ml
P-LAT (Tyr200
B Coll 3 Coll 10 CRP 3 CRP 10 -ve Coll 10 ug/ml
GAPDH
B Coll 3 Coll 10 CRP 3 CRP 10 -ve Coll 10 ug/ml
V220D Patient
Pan LAT 36-38kDa
B Coll 3 Coll 10 CRP 3 CRP 10 -ve Coll 10ug/ml
P-LAT (Tyr200)
B Coll 3 Coll 10 CRP 3 CRP 10 -ve Coll 10ug/ml
GAPDH
B Coll 3 Coll 10 CRP 3 CRP 10 -ve Coll 10ug/ml
K219N Patient
Pan LAT 36-38kDa
B Coll 3 Coll 10 CRP 3 CRP 10 -ve Coll 10ug/ml
P-LAT (Tyr200)
B Coll 3 Coll 10 CRP 3 CRP 10 -ve Coll 10ug/ml
GAPDH
(uM)
B PAR1 30 PAR1 100 ADP 30 ADP 100 -ve Coll 10ug/ml

Figure 1C

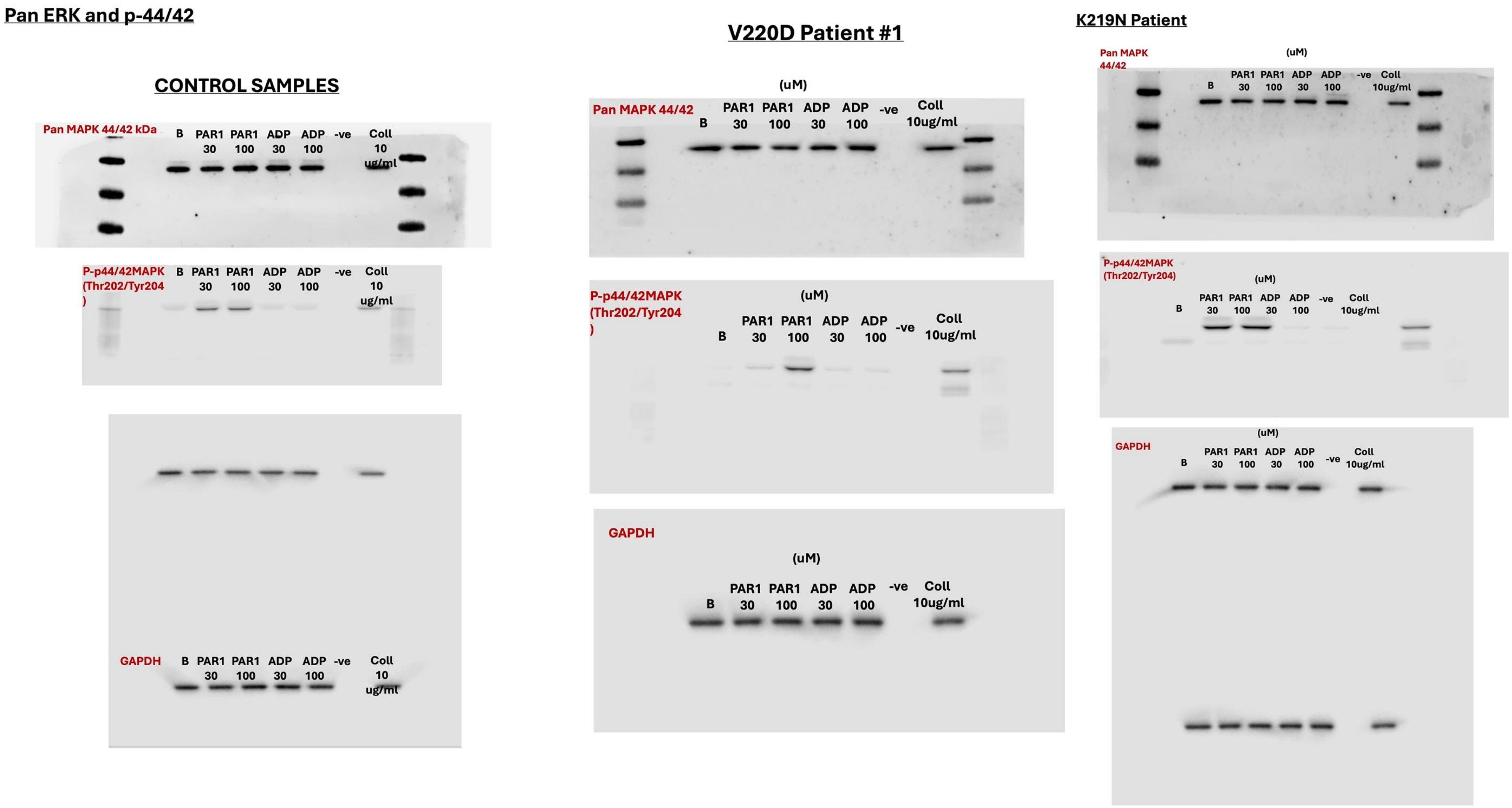
Pan ERK and p-44/42
CONTROL SAMPLES
Pan MAPK 44/42 kDa
B PAR1 30 PAR1 100 ADP 30 ADP 100 -ve Coll 10 ug/ml
P-p44/42MAPK (Thr202/Tyr204 )
B PAR1 30 PAR1 100 ADP 30 ADP 100 -ve Coll 10 ug/ml
GAPDH
B PAR1 30 PAR1 100 ADP 30 ADP 100 -ve Coll 10 ug/ml
V220D Patient #1
(uM)
Pan MAPK 44/42
B PAR1 30 PAR1 100 ADP 30 ADP 100 -ve Coll 10ug/ml
P-p44/42MAPK (Thr202/Tyr204 )
(uM)
B PAR1 30 PAR1 100 ADP 30 ADP 100 -ve Coll 10ug/ml
GAPDH
(uM)
B PAR1 30 PAR1 100 ADP 30 ADP 100 -ve Coll 10ug/ml
K219N Patient
Pan MAPK 44/42
(uM)
B PAR1 30 PAR1 100 ADP 30 ADP 100 -ve Coll 10ug/ml
P-p44/42MAPK (Thr202/Tyr204)
(uM)
B PAR1 30 PAR1 100 ADP 30 ADP 100 -ve Coll 10ug/ml
GAPDH
(uM)
B PAR1 30 PAR1 100 ADP 30 ADP 100 -ve Coll 10ug/ml

Figure 1D

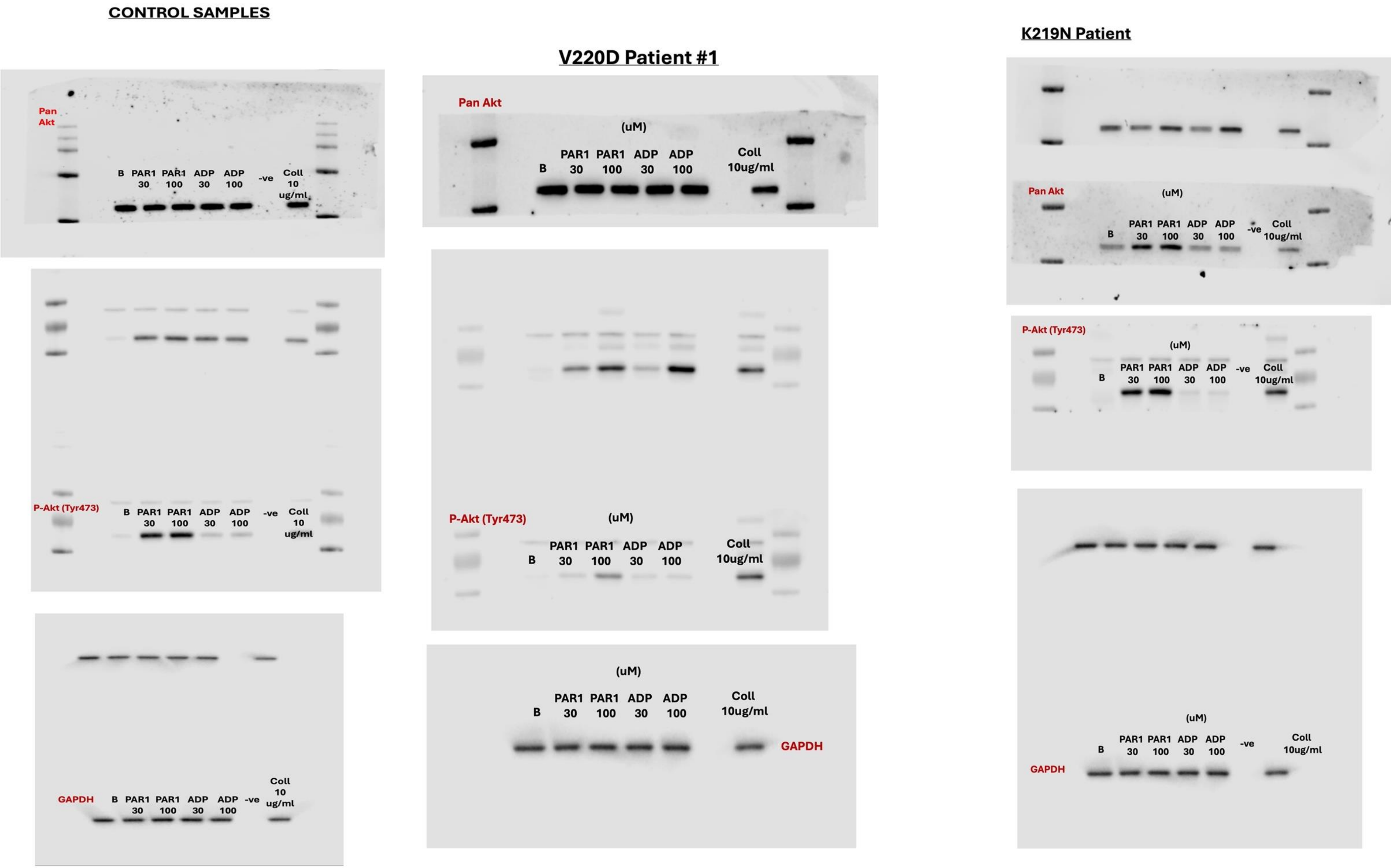
CONTROL SAMPLES
V220D Patient #1
K219N Patient
Pan Akt
B
PAR1 30
PAR1 100
ADP 30
ADP 100
-ve
Coll 10 ug/ml
P-Akt (Tyr473)
GAPDH
(uM)
Coll 10ug/ml

Supplemental Figure 6 A–B. Increased ribosomal protein S6 levels in *Slfn14*-deficient mouse and *SLFN14* mutant human platelets. (A) Western blot analysis of total S6 protein levels in platelets from *Slfn14*$^{+/+}$*;PF4-Cre* and *Slfn14*$^{fl/fl}$*;PF4-Cre* mice. (B) Western blot showing S6 protein levels in platelets from healthy controls (Con) and two *SLFN14* V220D variant patients. *GAPDH* served as a loading control.

S6

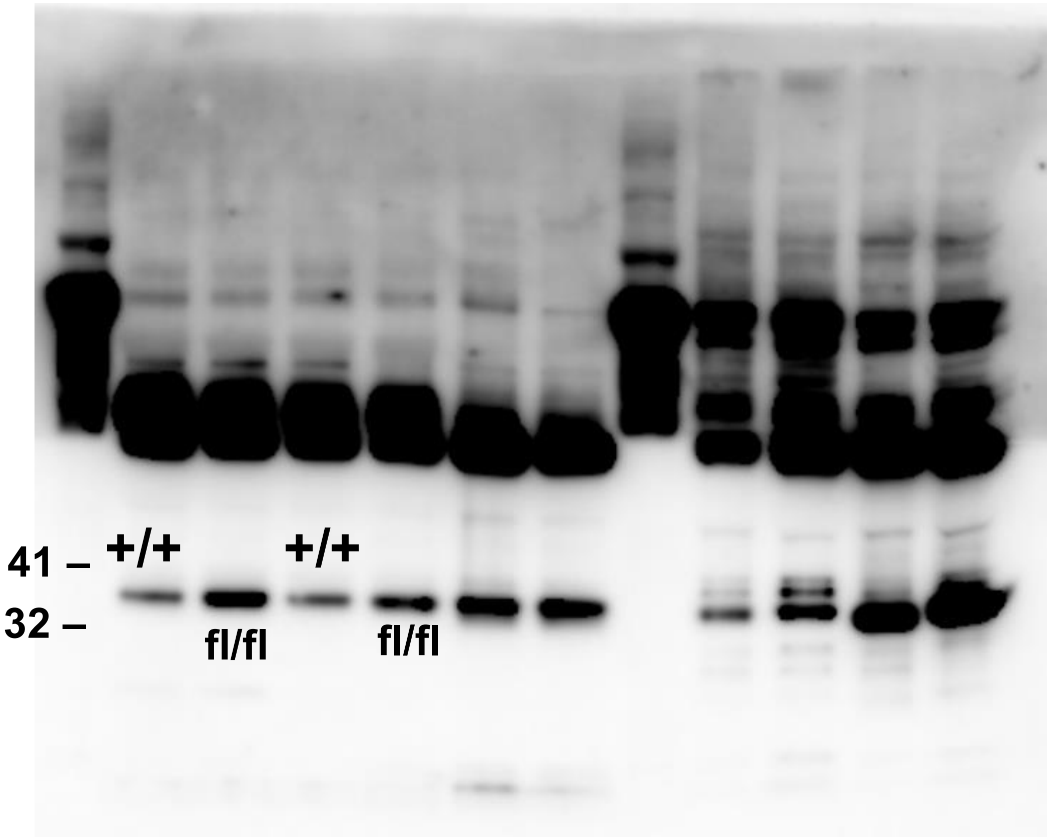


GAPDH

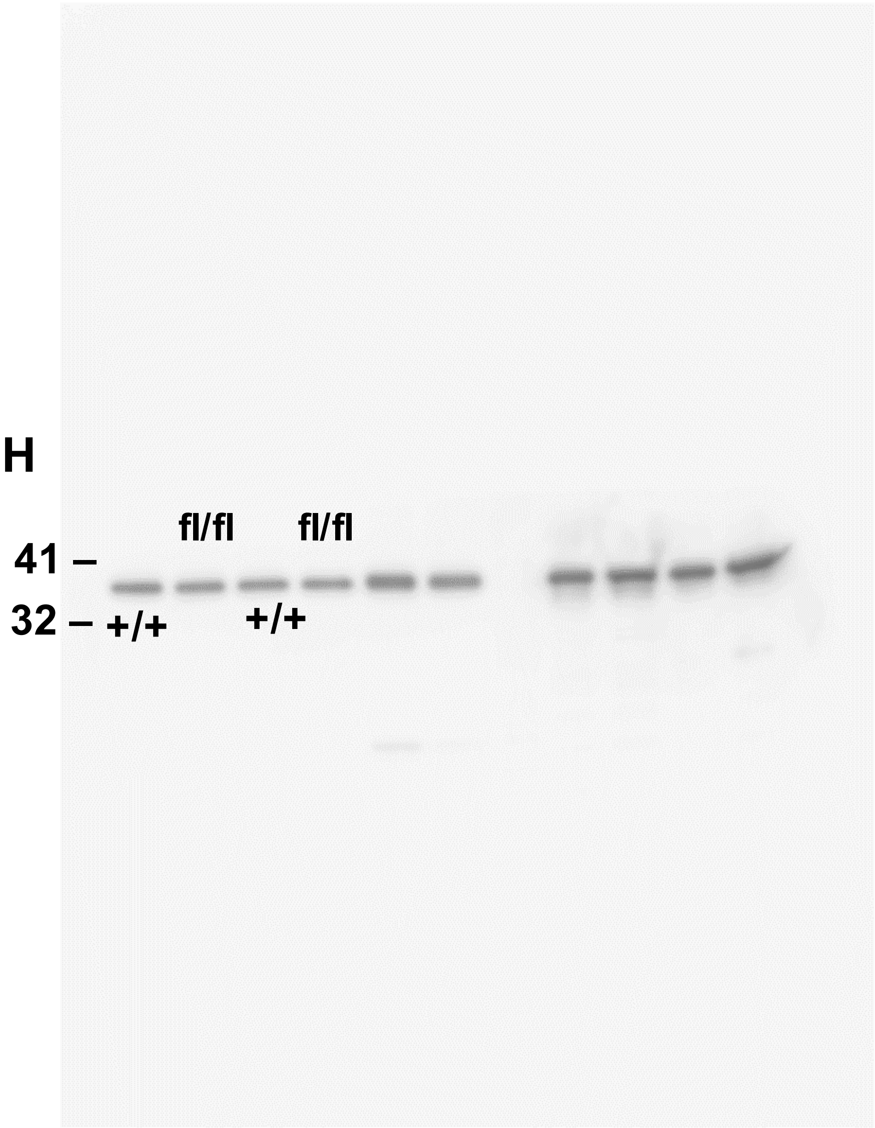

Supplemental Figure 6 A–B. Increased ribosomal protein S6 levels in *Slfn14*-deficient mouse and *SLFN14* mutant human platelets. (A) Western blot analysis of total S6 protein levels in platelets from *Slfn14*$^{+/+}$*;PF4-Cre* and *Slfn14*$^{fl/fl}$*;PF4-Cre* mice. (B) Western blot showing S6 protein levels in platelets from healthy controls (Con) and two *SLFN14* V220D variant patients. *GAPDH* served as a loading control.

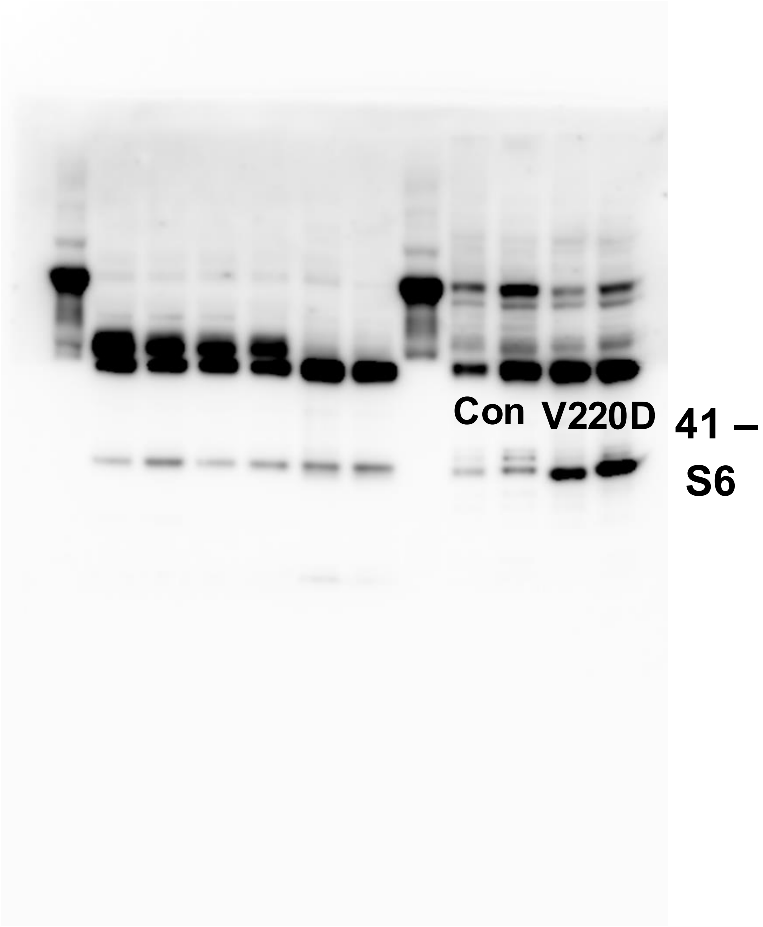


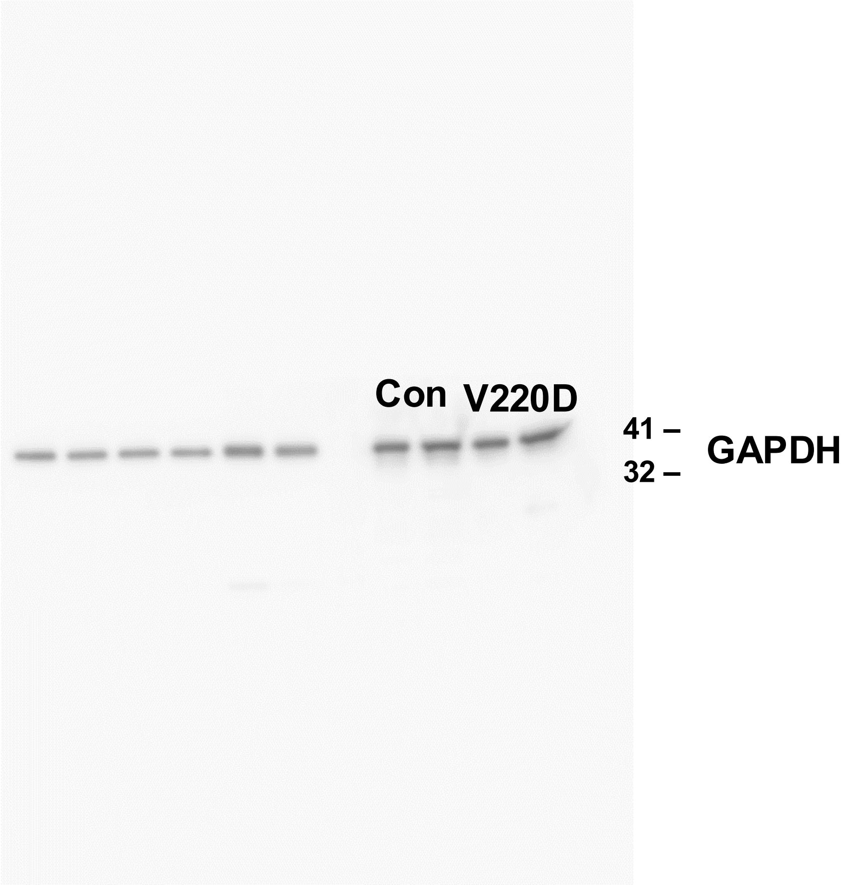

Supplement: Unedited blot and gel images [file jci-135-189100-s051.pdf]
